# An mTORC1 to HRI signaling axis promotes cytotoxicity of proteasome inhibitors in multiple myeloma

**DOI:** 10.1038/s41419-022-05421-4

**Published:** 2022-11-18

**Authors:** Odai Darawshi, Barbara Muz, Shiri Gershon Naamat, Bellam Praveen, Mohamed Mahameed, Karin Goldberg, Priya Dipta, Miriam Shmuel, Francesca Forno, Shatha Boukeileh, Hadas Pahima, Julia Hermann, Marc S. Raab, Alexandra M. Poos, Niels Weinhold, Chaggai Rosenbluh, Moshe E. Gatt, Wilhelm Palm, Abdel Kareem Azab, Boaz Tirosh

**Affiliations:** 1 Institute for Drug Research, The Hebrew University of Jerusalem, Jerusalem, Israel; 2 Department of Radiation Oncology, Washington University in St. Louis School of Medicine, St. Louis, MO USA; 3 Department of Genetics, The Hebrew University of Jerusalem, Jerusalem, Israel; 4 German Cancer Research Center (DKFZ), Heidelberg, Germany; 5 Department of Internal Medicine V, University Hospital Heidelberg, Heidelberg, Germany; 6 Hematology Dept. Hadassah Medical Center, The Hebrew University of Jerusalem, Jerusalem, Israel; 7 Department Biomedical Engineering, UT Southwestern, Dallas, TX USA; 8 Case Western Reserve University, Department of Biochemistry, Cleveland, OH USA

**Keywords:** Cancer metabolism, Apoptosis

## Abstract

Multiple myeloma (MM) causes approximately 20% of deaths from blood cancers. Notwithstanding significant therapeutic progress, such as with proteasome inhibitors (PIs), MM remains incurable due to the development of resistance. mTORC1 is a key metabolic regulator, which frequently becomes dysregulated in cancer. While mTORC1 inhibitors reduce MM viability and synergize with other therapies in vitro, clinically, mTORC1 inhibitors are not effective for MM. Here we show that the inactivation of mTORC1 is an intrinsic response of MM to PI treatment. Genetically enforced hyperactivation of mTORC1 in MM was sufficient to compromise tumorigenicity in mice. In vitro, mTORC1-hyperactivated MM cells gained sensitivity to PIs and hypoxia. This was accompanied by increased mitochondrial stress and activation of the eIF2α kinase HRI, which initiates the integrated stress response. Deletion of HRI elevated the toxicity of PIs in wt and mTORC1-activated MM. Finally, we identified the drug PMA as a robust inducer of mTORC1 activity, which synergized with PIs in inducing MM cell death. These results help explain the clinical inefficacy of mTORC1 inhibitors in MM. Our data implicate mTORC1 induction and/or HRI inhibition as pharmacological strategies to enhance MM therapy by PIs.

## Introduction

Multiple myeloma (MM) is the second most common hematological malignancy and represents approximately 20% of deaths from blood cancers [1, 2]. MM develops in the bone marrow (BM) and its growth, dissemination, therapy, and development of treatment resistance is directly affected by the interaction of MM cells with the BM tumor microenvironment (TME) [3, 4]. MM uniquely responds to proteasome inhibitors (PIs). Since their initial FDA approval in 2003, PIs have become the standard of care for MM and have significantly increased the survival and progression-free time of MM patients [5, 6]. However, the majority of MM patients relapse, while developing drug resistance and development of minimal-residual disease (MRD), which ultimately becomes lethal [3, 7, 8]. Resistance to PIs is primarily related to deregulated cell signaling pathways and modulation of cellular metabolism, for which interactions with the TME are important [4, 9, 10]. Hypoxia conditions develop in the MM microenvironment in correlation with tumor burden and directly promote MM recirculation and metastatic potential [11]. In addition, hypoxia is associated with the development of resistance to PIs [12]. In fact, a short exposure of MM to hypoxic conditions is sufficient to mediate resistance to PIs [13].

Although mTORC1 activity is upregulated in MM, mTORC1 inhibitors, including rapamycin, rapamycin analogs, and mTOR kinase inhibitors, are largely ineffective in MM, despite promising results in pre-clinical models [14]. The kinase mechanistic target of rapamycin (mTOR) resides in two complexes, mTORC1 and mTORC2. mTORC1 is a central coordinator of metabolism, which obtains inputs on nutrient, oxygen, ATP, and growth factor availabilities to adjust cellular metabolism, survival, and growth [15, 16]. mTORC1 activity is frequently elevated in cancer and promotes anabolic metabolism and cancer cell growth. However, under certain growth conditions, such as starvation conditions where cancer cells rely on proteins as an essential source for amino acids (AAs), mTORC1 suppresses tumor growth and mTOR inhibitors thereof paradoxically promote cellular survival and growth [17]. mTORC1 activity is negatively controlled by the tuberous sclerosis complex (TSC) [18], which integrates upstream inputs from growth factor signaling and stress pathways. Deletion of its subunits TSC1 or TSC2 leads to a constitutive activation of mTORC1 [19, 20]. mTORC1 also responds to amino acid (AA) availability [21]. In the amino acid-sensing pathway, mTORC1 is suppressed by the GATOR1 complex [22]. Thus, similar to the genetic ablation of the TSC, the removal of GATOR1 subunits, such as NPRL2, results in constitutive mTORC1 activation [23]. While mTORC1 activation is observed in various types of cancer, in MM, suppression of mTORC1 can facilitate tumor growth. For instance, the inhibitor of mTORC1, Deptor, is highly expressed in a subset of MM. Deptor downregulation induces mTORC1, and compromises MM survival [24, 25]. The proposed mechanism of death was attributed to reduced Akt activity, owing to a negative feedback loop through which mTORC1 suppresses PI3K-Akt activation [26].

The integrated stress response (ISR) is activated in response to various stress conditions that converge on the phosphorylation of the translation initiation factor eIF2α, by engaging either of the four eIF2α kinases: PKR, PERK, GCN2, or HRI. Phosphorylation of eIF2α limits the activity of eIF2B, which leads to a lower pool of the ternary complex, eIF2-GTP-Met-tRNA_i_^Met^, and hence a strong decrease in the initiation of protein translation. While the ISR confers a strong reduction in total protein synthesis, translation of a small subset of mRNAs is induced, most notably the transcription factor ATF4 [27]. Mild ISR activation is considered to be cytoprotective, by limiting stress-inducing pathways, preserving energy, and promoting autophagy. By contrast, persistent activation of the ISR leads to apoptosis. Thus, the role of the ISR in cancer can be both protective [28] and cytotoxic [29].

Here we show that mTORC1 suppression is a cell-intrinsic stress response of MM to PIs and hypoxia. Constitutive activation of mTORC1 compromises MM tumorigenicity and potentiates the toxicity of PIs and hypoxia. mTORC1-activated MM cells develop mitochondrial stress in the presence of PIs. As a consequence, cells strongly activate the ISR through HRI. HRI-deficient MM cells acquire susceptibility to PIs, suggesting the ISR as a pro-survival response. We further identify the PKC activator phorbol myristate acetate (PMA) as an mTORC1-activating drug, which sustained mTORC1 activity in the presence of PIs and synergized with the PI ixazomib to kill MM cells. Deletion of HRI further enhanced the sensitivity to PIs and to PI/PMA combination in wt MM. Similar results were obtained in glioblastoma cells. We propose that a combination of PIs with mTORC1 activators or HRI inhibitors can improve the clinical efficacy of PIs in MM and in other cancers, such as glioblastoma.

## Results

### Hypoxia and proteasomal inhibition independently cause mTORC1 suppression in MM

**Fig. 1:**
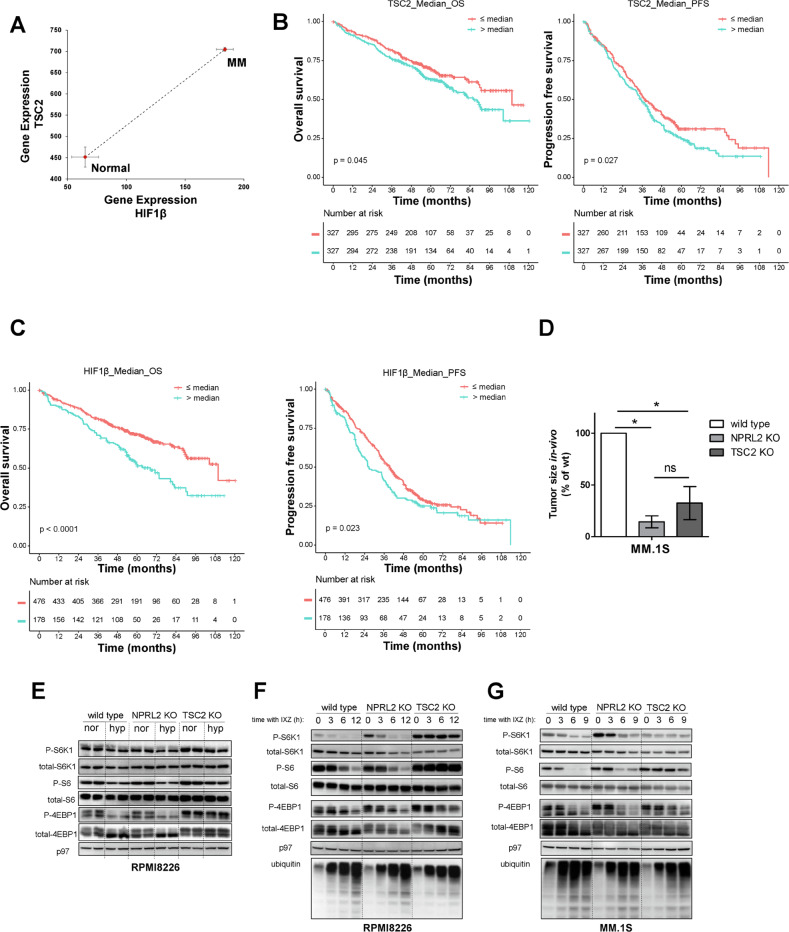
Hypoxia and proteasomal inhibition independently cause mTORC1 suppression in MM. **A** A relative correlation between mRNA expression level of TSC2 and HIF1β in CD138 + bone marrow plasma cells from healthy subjects (n = 22) and newly diagnosed MM patients (*n* = 559). Axis units are arbitrary. Error bars represent S.E.M. **B**, **C** Shown are Kaplan–Meier plots for the overall survival (OS) and progression-free survival (PFS) of MM patients with differential expression of TSC2 (**B**) and HIF1β (**C**) from GMMG HD4 and MM5 trials. **D** Subcutaneous tumor growth of wt, NPRL2 KO, and TSC2 KO MM.1S cells into NCG mice (*n* = 4). Shown is the average relative tumor weight ± S.E.M. and the asterisk represents *p* < 0.05 of unpaired two-tailed student’s *t*-test between wt and KO. ns, not significant. **E** Cells were cultured in fresh media for 4 h in either normoxia or hypoxia. mTORC1 response was assessed by immunoblotting of its downstream phosphorylated and total effectors, S6K1, S6, and 4E-BP1. p97 was used to confirm equal protein loading in each lane. Shown are technical duplicates in each condition of a typical experiment of three repetitions. **F**, **G** Cells were cultured in fresh media for 16 h, then treated with IXZ [32 nM]. Following the treatment, cells were collected for immunoblotting in increasing time points as indicated. Shown are immunoblots for downstream effectors of mTORC1. Ubiquitin immunoblot was used to assess proteasomal inhibition. Shown are a typical immunoblots out of three independent experiments. nor, normoxia. hyp, hypoxia, IXZ, ixazomib.

We previously reported that hypoxia develops in the MM microenvironment in correlation with tumor burden and directly promotes MM recirculation and metastatic potential [11]. Hypoxia was implicated in the development of resistance to PIs by multiple mechanisms [12, 30], and the pre-conditioning of MM under hypoxia demonstrates a direct causative connection to resistance to PIs in vitro and in vivo [13]. ARNT/HIF-1β is the cofactor and nuclear translocator for hypoxia-induced transcription factor, HIF-1α [31]. Expression of HIF-1β is correlated to drug resistance and poor prognosis in MM [32]. To examine if attenuation in mTORC1 activity is associated with the development of hypoxia in the TME, we correlated the expression of TSC2 and HIF-1β in cohorts of normal bone marrow plasma cells and MM gene expression profiles. A clear positive correlation was observed between TSC2 and HIF-1β (Fig. 1A), suggesting that suppression of mTORC1 and development of hypoxia are connected in MM, and simultaneously develop in the course of the disease trajectory. This is opposite to what has been shown for TSC2 expression in acute leukemia [33]. To examine directly the correlation between TSC2 expression and MM prognosis, we analyzed clinical data of the GMMG HD4 and MM5 trials [34]. A higher expression of TSC2 was correlated with a worse overall survival and progression-free survival (Fig. 1B). A similar correlation was observed for HIF-1β (Fig. 1C). To examine the relationship of MM tumorigenicity and mTORC1, we constitutively activated mTORC1 by genetically ablating the negative upstream regulators, TSC or GATOR1 in RPMI8226 and MM1.S cells using CRISPR/Cas9-mediated gene editing for TSC2 and NPRL2 (Fig. S1A, B). Despite a similar growth in vitro (not shown), when the cells were implanted subcutaneously to NSG mice, tumor development was faster for control MM1.S than TSC2 and NPRL2 KO variants (Fig. 1D). Thus, constitutive activation of mTORC1 antagonizes MM growth in vivo.

Next, we examined the cellular response of MM to hypoxia and proteasome inhibitors. Hypoxia resulted in a marked suppression of mTORC1 activity in control RPMI8226 cells. To establish the relevance of mTORC1 inactivation in response to PI or hypoxia, we investigated cells with constitutively activated mTORC1. Deletion of NPRL2 did not affect mTORC1 response, while TSC2 provided a partial resistance to hypoxic conditions (Fig. 1E). Similar results were obtained for MM1.S cells (not shown). Next, we examined the response of mTORC1 to proteasomal inhibition. Exposure of MM cells to the PI ixazomib (IXZ) for 6 h resulted in a marked suppression of mTORC1 activity. mTORC1 inactivation was blocked by TSC2 deletion. By contrast, deletion of NPRL2, though compromising in vivo growth, had a minimal effect on the mTORC1 response to IXZ (Fig. 1F). A similar response to IXZ was observed in MM1.S cells (Fig. 1G). Analyses of the GMMG trials for NPRL2 did not show a significant correlation with MM prognosis (Fig. S2). Thus, in MM, mTORC1 activity is intrinsically sensitive to proteasomal inhibition and hypoxia in a mechanism that depends on TSC2. These results are consistent with the function of the TSC to communicate hypoxia and other stress signals to mTORC1.

Based on extensive preclinical data, the brain tumor glioblastoma was suggested to be sensitive to marizomib, a PI that crosses the blood-brain barrier [35]. Despite initial promise, marizomib failed to improve survival in clinical trials when combined with the standard of care, the alkylating agent temozolomide and radiochemotherapy [36]. We examined mTORC1 activity in the glioblastoma cell line GL261. A concentration of 2.5 µM of IXZ was sufficient to strongly reduce mTORC1 activity (Fig. S1C). mTORC1 activity in several other tumor cell lines, including melanoma MEL624, was not affected by IXZ (Fig. S1D). For other cells, such as pancreatic cancer (MIA-Paca2), suppression of mTORC1 required higher concentrations (Fig. S1E). We conclude that mTORC1 suppression by PIs is a response of a subset of cancers, including MM and glioblastoma, which are hyper sensitive to PIs.

### Hyperactive mTORC1 sensitizes MM to PIs independently of AKT and autophagy

**Fig. 2:**
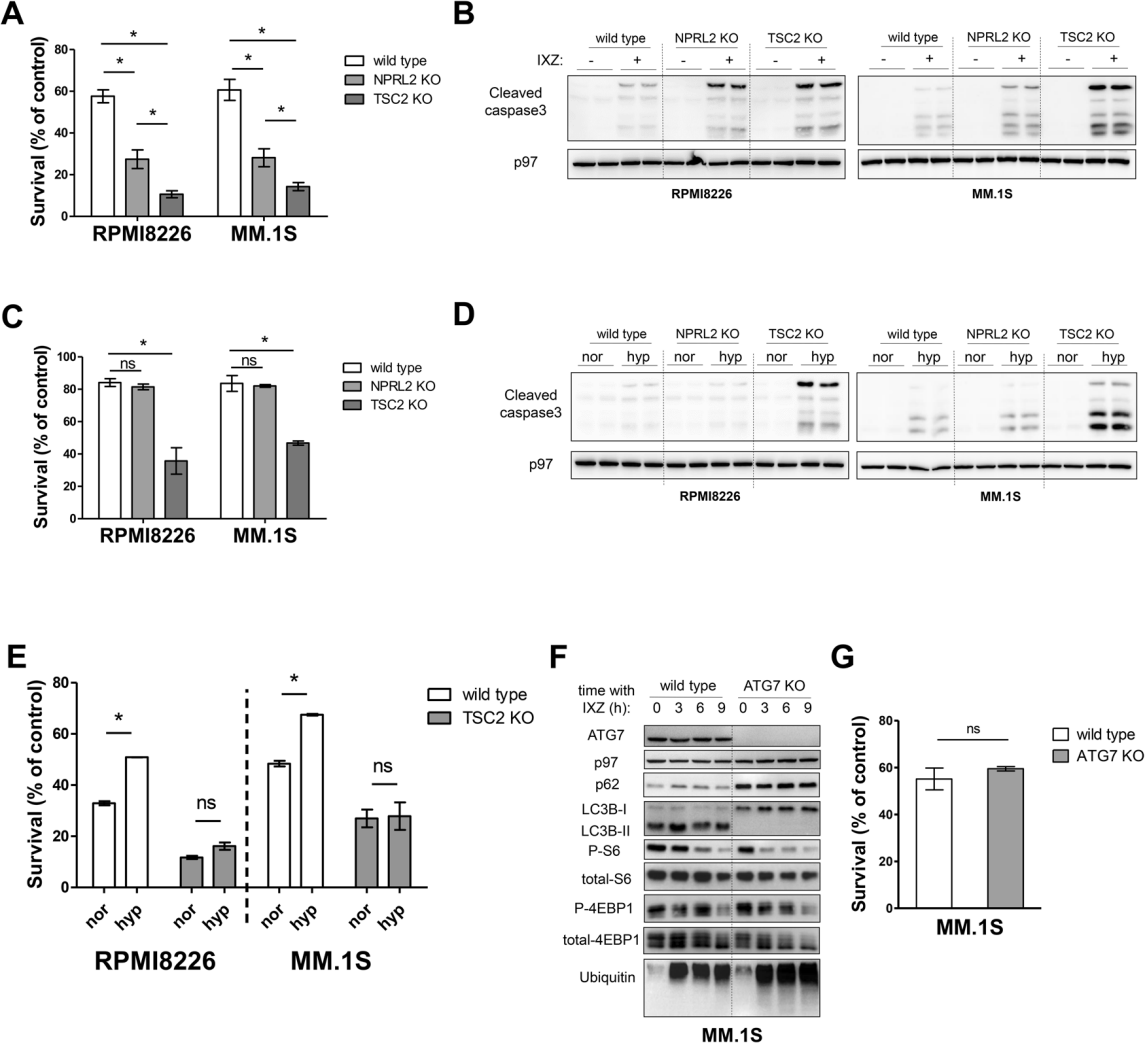
Hyperactive mTORC1 sensitizes MM to PIs independently of autophagy. **A** MM cells were treated with IXZ [20 nM] for 48 h. Shown is average relative viability of three independent experiment ± S.E.M., *p < 0.05 of unpaired two-tailed student’s *t*-test. **B** Cells were treated as described in (**A**) followed by immunoblotting analysis of cleaved caspase3 for apoptosis assessment and p97 as a loading control. Shown is a technical duplicate for each condition. **C** MM cells were incubated in hypoxia for 48 h. Shown is average relative viability of three independent experiment ± S.E.M., *p < 0.05 of unpaired two-tailed student’s *t*-test. **D** Cells were incubated as described in (**C**) followed by immunoblotting analysis of cleaved caspase3 for apoptosis assessment and p97 as a loading control. Shown is a technical duplicate for each condition. **E** MM cells were treated with IXZ [40 nM] for 24 h in either normoxia or hypoxia as indicated. Shown is average relative viability of three independent experiment ± S.E.M., *p < 0.05 of unpaired two-tailed student’s *t*-test. ns, not significant. **F** A time-response analysis of mTORC1 and autophagy activities following IXZ [32 nM] treatment in wt versus ATG7 KO MM.1S cells. Inhibition of autophagy was confirmed by immunoblotting for p62 and LC3BI/II proteins. Shown is a typical immunoblots out of three independent experiments. **G** MM cells were treated with IXZ [20 nM] for 48 h. Shown is the average relative viability of three independent experiment ± S.E.M., ns, not significant.

To examine whether the reduction in mTORC1 activity contributed to attenuated toxicity of PIs, we treated MM cells, in which mTORC1 is constitutively activated by deletion of TSC2 or NPRL2, with IXZ. Both TSC2 and NPRL2 KO MM cells were more sensitive to IXZ than control cells (Fig. 2A). The increased sensitivity was correlated with higher levels of cleaved caspase 3, consistent with enhanced apoptosis (Fig. 2B). Of note, TSC2 KO were more sensitive to IXZ than NPRL2 KO cells, which correlated with their ability to sustain mTORC1 activity at higher levels. TSC2 KO cells were also hypersensitive to hypoxia relative to NPRL2 KO and control cells, which was congruent with the similar reduction of mTORC1 activity in control and NPRL2 KO cells (Fig. 2C). This was also reflected in the levels of cleaved caspase 3 (Fig. 2D). Exposure of MM to hypoxic conditions is sufficient to induce resistance to PIs [12]. Importantly, the induction of resistance to PIs by hypoxia, seen in wt cells, was not apparent in TSC2 KO cells (Fig. 2E). Overall, this suggests that the decrease in mTORC1 activity in response to PIs and hypoxia is cytoprotective and required to establish resistance.

mTORC1 is a key negative regulator of autophagy, a pathway that promotes survival under various stress conditions [37]. Autophagy also promotes cell survival in response to PI treatment, conceivably by delivering proteins to the lysosome as an alternative degradation pathway. Consistently, hydroxychloroquinone, which inhibits lysosomal degradation, enhances the toxicity of the PI bortezomib BTZ [38, 39]. To test whether mTORC1 hyperactivation sensitized MM cells to PIs by blocking autophagy, we generated ATG7 KO cells in MM1.S, which abolished autophagy, as determined by the absence of LC3B-II (Fig. 2F). The sensitivity to IXZ was similar in wt and ATG7 KO MM (Fig. 2G). The similar response of glioblastoma and MM to PIs prompted us to examine whether in glioblastoma cells the sensitivity to PIs is influenced by autophagy. Deletion of TSC2 in the glioblastoma GL261 cells enhanced their sensitivity to IXZ, while deletion of ATG7 did not, similar to MM (Fig. S3A–D). We conclude that mTORC1 activation by TSC2 deletion sensitizes MM to PIs in an autophagy-independent manner.

The mTORC1 inhibitor Deptor is highly expressed in a subset of MM, those harboring cyclin D1/D3 or c-MAF/MAFB translocations [26]. Small molecules that direct Deptor for degradation or genetic suppression of Deptor promote MM cell death. Deptor inhibition synergizes with BTZ for MM therapy [25], reinforcing the effect of mTORC1 activation in the sensitivity to PIs. The underlying mechanism implicated the enhancement of the negative feedback between mTORC1 and PI3K signaling [40], which results in a strong inhibition of AKT. Analysis of AKT activity in MM1.S and RPMI8226 cells in which mTORC1 was induced by NPRL2 or TSC2 deletion did not show a significant difference in AKT phosphorylation in (Fig. S3E, F). Moreover, the inactivation of mTORC1 by depletion of amino acids (AAs) did not significantly affect AKT activity, indicating that in these MM cells, the mTORC1/PI3K feedback is not dominant (Fig. S2E, F). We conclude that suppression of AKT is not the major factor responsible for promoting sensitivity to PIs in mTORC1 overactive cells.

### mTORC1-activated MM cells develop a mitochondrial stress and induce the ISR by HRI in response to PIs

**Fig. 3:**
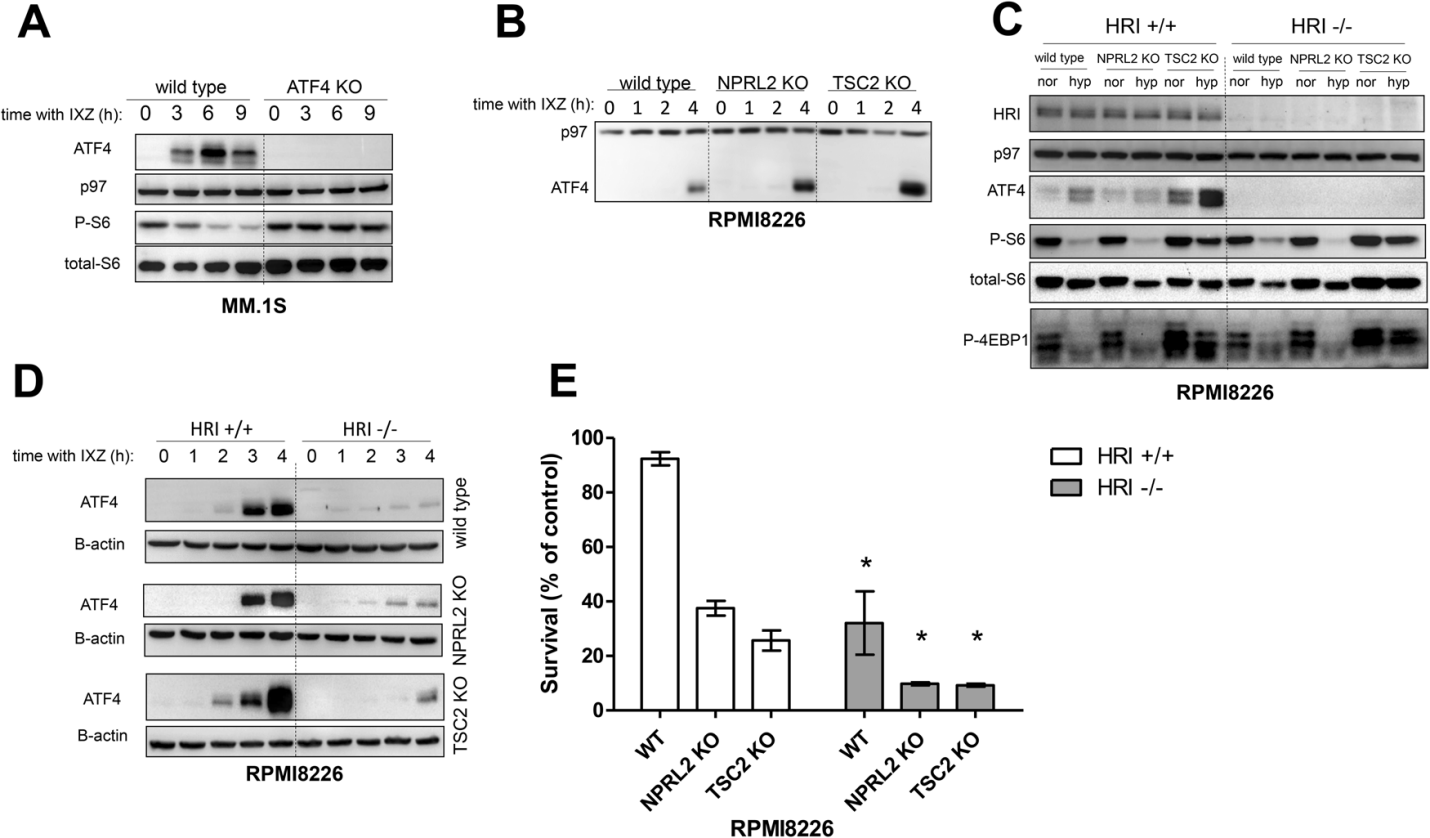
mTORC1-activated MM cells mount an ISR by the activation of HRI in response to PIs and hypoxia. **A** Shown is a typical response of mTORC1 in wt and ATF4 KO MM.1S cells following IXZ [32 nM] treatment at the indicated time points. **B** ATF4 induction was assessed by imunoblotting following IXZ [32 nM] treatment. **C** ATF4 induction and mTORC1 activity were assessed following hypoxia for 4 h for wt and KO HRI cells as indicated. **D** ATF4 induction was measured following IXZ [32 nM] treatment in a time-dependent manner in both wt HRI and KO HRI cells. **E** Cells were treated with IXZ [10 nM] for 48 h. Shown is average relative viability of three independent experiment ± S.E.M., *p < 0.05 of unpaired two-tailed student’s *t*-test between wt HRI and KO HRI of each indicated variant.

A genome-wide genetic screen in 293T cells identified the transcription factor ATF4 as central to the supression of mTORC1 by multiple downstream targets, in particular Sestrin2 and Redd1 [41]. Consistently, deletion of ATF4 in MM cells prevented the suppression of mTORC1 in response to IXZ (Fig. 3A). Comparison of ATF4 expression of control and mTORC1-activated MM in response to IXZ showed that ATF4 expression correlated with mTORC1 induction (TSC2 KO > NPRL2 KO > wt) (Fig. 3B). Induction of ATF4 expression is a hallmark of the ISR, which is controlled by the phosphorylation of eIF2α. However, it was suggested that mTORC1 enhances ATF4 expression in an eIF2α phosphorylation-independent manner, when activated by growth factor signaling [42]. Thus, we examined whether the induction of ATF4 is mediated by eIF2α kinases. Supporting evidence exists for either PERK, GCN2 and HRI. The eIF2α kinase PERK transduces ER stress, which can be a consequences of proteasomal inhibition. Thus, we tested whether PERK is involved in the hyperactivation of the ISR in mTORC1-activated cells. Inhibition of PERK did not abolish the induction of ATF4 in response to IXZ. Rather, ATF4 induction was increased by PERK inhibition and was completely blocked by rapamycin treatment (Fig. S4A). PIs cause a depletion of free AAs, which activates the eIF2α kinase GCN2 [43]. In the presence of a GCN2 inhibitor, mTORC1-activated MM induced a similar level of ATF4 in response to IXZ (Fig. S4B). Due to the lack of a specific inhibitor to HRI, we generated HRI KO in control, TSC2, and NPRL2 KO cells. Deletion of HRI eliminated ATF4 induction by hypoxia (Fig. 3C). Similarly, deletion of HRI strongly diminished induction of ATF4 in response to IXZ (Fig. 3D). This suggests that mTORC1 activity in response to PIs predominantly promotes the activation of HRI. Since the ISR can have both protective and pro-death roles, depending on intensity and duration, we assayed the toxicity of IXZ in HRI-proficient and deficient cells. For all genetic variants, deletion of HRI promoted IXZ toxicity (Fig. 3E). This suggests that the ISR is protective and HRI a potential target in MM. Importantly, deletion of HRI in other cell types, glioblastoma, 293T, pancreatic carcinoma and melanoma, resulted in increased toxicity of IXZ (Fig. S5), suggesting that a potential HRI inhibitor can be used in combination with PIs for additional tumors.

**Fig. 4:**
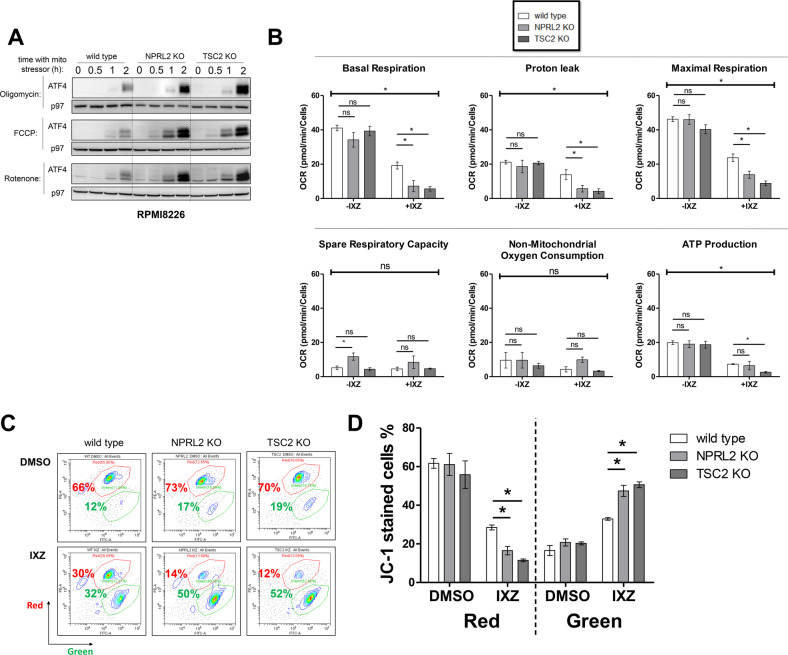
mTORC1-activated MM cells generate a mitochondrial stress in response to mitotoxins and PIs. **A** ATF4 induction was monitored following oligomycin, FCCP and rotenone (each 1 µM) treatment in time-escalated manner as indicated. **B** Average OCR measurements are shown following treatment with either DMSO (-IXZ) or IXZ (+IXZ) [20 nM for 16 h], error bars represent ±S.E.M., *p < 0.05 of unpaired two-tailed student’s *t*-test. ns, not significant. **C** Contour plots of JC-1 stained cells following treatment with either DMSO or IXZ [20 nM for 24 h] are shown and each represents a typical result out of three independent experiments. Red gate corresponds to cells with ‘healthy’ mitochondrial membrane potential and green gate corresponds to cells with ‘unhealthy’ mitochondrial membrane potential. **D** Column graph represents the average of three independent experiments described in (**C**), ±S.E.M., *p < 0.05 of unpaired two-tailed student’s *t*-test.

HRI is activated by mitochondrial stress [44, 45]. To assess whether mitochondrial stress is the underlying reason for the activation of HRI when mTORC1 is induced, we treated the cells with three mitochondrial stress inducers, oligomycin (F_1_F_0_-ATPase complex inhibitor), rotenone (inhibitor of the mitochondrial complex I) and FCCP (uncoupler). For either mitostressor, the expression of ATF4 was higher, in correlation with mTORC1 induction (Fig. 4A). Next, we monitored mitochondrial respiration and membrane potential in control and mTORC1-activated cells in the presence of IXZ. In the absence of IXZ, mitochondrial respiratory parameters were not significantly different between control and NPRL2 KO or TSC2 KO MM. In the presence of IXZ, basal respiration, proton leak, maximal respiration and ATP production were reduced in both NPRL2 KO and TSC2 KO MM compared to control (Fig. 4B). ATP production in NPRL2 KO or WT cells was similar. A significant reduction was observed in TSC2 KO cells. As both maximal and basal respiration were reduced to same extent after the treatment, the spare respiratory capacity was not significantly changed. Non-mitochondrial respiration was not affected by IXZ treatment, indicating that other oxygen consuming metabolic processes were not affected by the treatment. This was accompanied by a decrease in mitochondrial membrane potential, as determined by JC-1 red to green shift (Fig. 4C, D). Thus, mTORC1 activation predisposes MM cells to develop a mitochondrial stress in response to PIs.

### The phorbol ester PMA is an effective mTORC1 inducer that facilitate PI toxicity

Next we searched for a pharmacological approach that can recapitulate the TSC2 deletion, aiming to identify drugs that synergize with PIs by preventing mTORC1 suppression. Several molecules have been shown to induce mTORC1. A large number of reports demonstrate that MHY1485 increases mTORC1 activity and blocks autophagy [46]. The addition of MHY1485 did not affect mTORC1 activity in MM and did not prevent the suppression in mTORC1 activity upon amino acid depletion (Fig. S6A). We also tested the AMPK inhibitor Dorsomorphin (a.k.a compound C), which under certain stress conditions activates mTORC1. Dorsomorphin exerts its function by multiple mechanisms. For instance it promtes cell death of glioblastoma, despite reducing mTORC1 activity [47]. Similarly to this observation, by itself, Dorsomorphin reduced, rather than induced, mTORC1 activity in RPMI8226 cells, despite blocking AMPK (Fig. S6B). In addition, we used the leucine analouge, NV-5138, however it did not sustained mTORC1 activity under AA starvation and/or IXZ treatment (Fig. S6C and D).

**Fig. 5:**
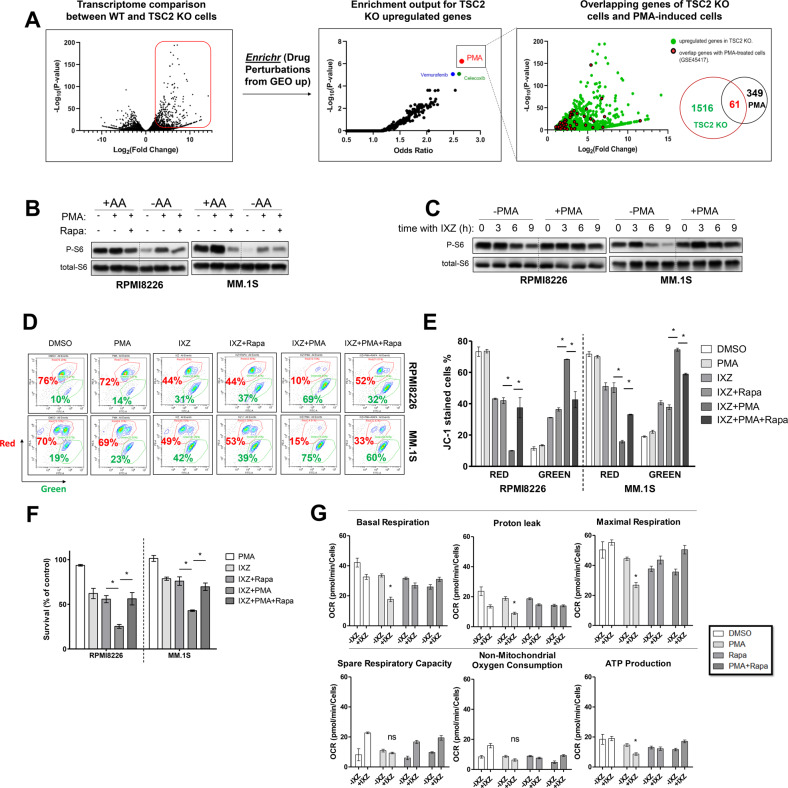
The phorbol ester PMA is an effective mTORC1 inducer that facilitate PI toxicity. **A** Shown is a volcano plot representing transcriptome comparison between wt RPMI8226 cells (*n* = 3 independent samples) and TSC2 KO RPMI8226 cells (*n* = 2 independent samples). Upregulated genes in TSC2 KO cells (*n* = 1516, FC > 2, *p* < 0.05) are shown in red rectangle. Enriched genes were analyzed using ‘Drug Perturbations from GEO up’ as a reference library. Enrichment output is shown in the middle volcano plot. Overlapping genes are shown in the right graph and correspond to TSC2 KO upregulated genes set. **B** MM cells were treated with PMA [125 nM] alone or in combination with rapamycin [50 nM] for 2 h in the presence and absence of AA, as indicated. Shown are a typical immunoblots out of three independent experiments. **C** MM cells were treated with IXZ [32 nM] alone or in combination with PMA [125 nM]. Treatment was performed in a time-dependent manner, as indicated. Shown are a typical immunoblots out of three independent experiments. **D** MM cells were treated for 24 h with PMA [125 nM], IXZ [15 nM], rapamycin [50 nM], as indicated. Contour plots of JC-1 stained cells following the treatment are shown and each represents typical result out of two independent experiments. Red gate corresponds to cells with ‘healthy’ mitochondrial membrane potential and green gate corresponds to cells with ‘unhealthy’ mitochondrial membrane potential. **E** Column graphs corresponding to the experiment described in (**D**), error bars represent S.E.M., *p < 0.05 of unpaired two-tailed student’s *t*-test. **F** MM cells were treated for 48 h with PMA [125 nM], IXZ [15 nM], rapamycin [50 nM], as indicated. Shown is average relative viability of three independent experiment ± S.E.M., *p < 0.05 of unpaired two-tailed student’s *t*-test. **G** Average OCR measurements are shown following treatment with DMSO, PMA [125 nM], rapamycin [50 nM] and IXZ [10 nM] for 12 h, error bars represent ±S.E.M., *p < 0.05 of unpaired two-tailed student’s *t*-test. ns, not significant.

To identify potential small molecules that activate mTORC1, we performed an RNAseq transcriptome profiling for TSC2 KO and control RPMI8226 cells. This profiling identified 1516 significantly upregulated genes (FC > 2, *P* < 0.05) in TSC2 KO cells. When this list was compared to transcriptome data of drug-treated cells using Enrichr [48], the compound phorbol-12-myristate-13-acetate (PMA) stood out as the most significant one (Fig. 5A). PMA is a PKC activator that pharmacodynamically mimicks the interaction of PKC with diacyl glycerol. Simirlarly to diacyl glycerol, PMA activates classical and non-classical PKC isoforms [49, 50]. In HEK293 cells, sustained PKC activity conferred by PMA at 100 nM, activates mTORC1 [51], primarily by promoting the activity of PKCη [51]. We evaluated the effect of PMA on mTORC1 in MM cells alone and in combination with IXZ. At 125 nM PMA induced phosphorylation of the mTORC1 pathway target S6 in MM1.S and RPMI8226 cells (Fig. 5B). Importantly, PMA prevented mTORC1 suppression by PI treatment (Fig. 5C). Thus, we tested whether PMA synergized with PIs in decreasing MM cell viability. JC-1 red fluorescence was reduced by the combination of IXZ and PMA in both MM cell types, similar to combination of PMA and genetically activated mTORC1. The decrease in JC-1 fluorescence was partially reversed by rapamycin, consistent with a role for mTORC1 in mitochondrial stress generation (Fig. 5D, E). Analysis of cell viability by flow cytometry demonstrated that PMA alone was not toxic, in combination of PMA with IXZ led to elevated cell death. Rapamycin abolished this additive effect of PMA (Fig. 5F). Overall, this suggests that PMA recapitulates the genetic activation of mTORC1 as a potentiator of IXZ anti-MM activity. We then tested whether mitochondrial respiration is compromised in the presence of PMA and IXZ, as was seen for the TSC2 KO MM cells. We treated WT cells with DMSO, PMA, rapamycin, and a PMA/rapamycin combination in the presence and absence of IXZ. As predicted, the combined IXZ and PMA treatment phenocopied the effect of mTORC1-activated MM with PI. The only treatment that significantly affected mitochondrial respiration was the PMA + IXZ, indicating that the combined treatment has an additional effect than each treatment alone. The addition of rapamycin reversed effect. PMA + IXZ treatment reduced basal respiration, proton leak, maximal respiration and ATP production, while both spare respiratory capacity and non-mitochondrial respiration were not significantly different from the other treatments. Of note, the two additional drugs that were highlighted by Enrichr, vemurafenib or celecoxib, were not efficient in activating mTORC1 alone and did not prevent the suppression of mTORC1, when combined with IXZ (Fig. S7A–C). Since HRI deletion enhanced IXZ toxicity in both wt and mTORC1-activated MM cells, we applied IXZ + PMA combination on HRI KO MM cells and observed enhanced toxicity under this combination (Fig. S7D). Furthermore, as glioblastoma responded similarly to MM to PI treatment, we tested the effect of PMA and IXZ on GL261. The addition of PMA to the glioblastoma cells prevented the suppression of mTORC1 by IXZ (Fig. S8A). Examination of viabality showed that PMA was not toxic on its own, but potentiated the cytotoxic effect of IXZ, and inhibition of mTORC1 partially rescued the cells (Fig. S8B). These findings suggest pharmacological stimulation of mTORC1 as a promising stragtegy to improve the efficacy of PIs in cancer treatment.

To assess whether mTORC1 activity has a clinical significance in MM therapy, we analyzed a small number of bone marrow aspirates, obtained at the time of diagnosis for mTORC1 activity using intracellular staining for P-S6. as a readout. When mTORC1 activity was correlated with resistance to BTZ, as develops following years of treatmet, a higher mTORC1 activity was scored for BTZ sensitive patients (Fig. S9). Though more samples are required, we suggest that mTORC1 activity can predict the development of resistance to BTZ.

## Discussion

Resistance to PIs in MM patients is a slow process driven by selection. In the vast majority of MM patients, resistance is not mediated by conventional mechanisms, such as upregulation of the efflux pumps [52], or mutations in the drug target, in this case the proteasome β5 subunit [52]. Rather, resistance to PIs is primarily associated with deregulated signaling and cellular metabolism [53]. A few studies implicated the mitochondria as a modulator of PI toxicity [54]. A recent study suggested that resistance to PIs is facilitated through a lower electron transport chain (ETC) [55]. Though mTORC1 activity is suppressed when ETC is inhibited, the relevance of mTORC1 to resistance to PIs was not addressed directly in this study. Here we underscore a cellular connection between mTORC1 and mitochondrial stress, which becomes apparent under treatment with PIs. While generally promoting cancer growth, in this context, mTORC1 compromises cell viability by promoting anabolic activities which consequently impart various stress conditions, including proteotoxic stress and ROS. When mTORC1 activity is enforced, adaptation to stress is impaired. We therefore propose that hyperactivation of mTORC1 can be exploited for therapy, when judiciously combined with stress inducing drugs.

We show that mTORC1 suppression in response to PIs is intrinsic and occurs within hours (Fig. 1). Importantly, mTORC1 inactivation provides protection from developing mitochondrial stress following exposure to PIs or hypoxia. When mTORC1 inactivation is prevented, genetically by TSC2 deletion or pharmacologically by treatment with PMA, MM cells succumb more readily to PIs and hypoxia (Figs. 2, 5). We show that mitochondria respond to PIs by activating HRI, which mediates a negative feedback response to suppress mTORC1 activity by promoting ATF4 expression and elicit a cytoprotective response (Fig. 3). Deletion of HRI enhanced PI toxicity in control and in TSC2 KO MM cells, suggesting that the negative feedback to mTORC1, which relies on ATF4 and TSC2, is not the only pro-survival mechanism downstream to HRI. While the function of HRI in fetal hemoglobin synthesis in human erythroid cells is well understood [56], the effects of HRI on cancer development and response to therapy have not been thoroughly investigated. HRI expression is ubiquitous, not restricted to erythrocytes, and its importance for cancer has been sporadically highlighted in the context of drug treatmets [57]. For instance, HRI inhibition by shRNA-mediated silencing enhanced the toxicity of the PI bortezomib in pancreatic cancer [58]. HRI is a key mediator of cell survival in prostate cancer when treated with BH3 mimetics [59]. Taken together with our data in MM and glioblastoma, this suggests a general role of HRI in cancer suseptibility to stress inducing drugs, such as PIs.

Because sensitivity to PIs is similar in ATF4 positive and negative MM (not shown), downstream to HRI, we suggest that phosphorylated eIF2α activates additional pro-survival response independently of ATF4. Little is known about the translation-independent biologial significance of phosphorylated eIF2α. One possibiity is a biophysical role of stress granules, which their biogenesis is dependent on eIF2α phosphorylation, but may mediate adaptations to stress regardless of translation regulation. A role in resistance to PIs has been shown [60]. Moreover, since stress granules are induced by mTORC1 [61], we suggest that HRI activation may be a general nexus to coordinate survival adaptation to PIs by ATF4-dependent and independent mechanisms. We propose that inhibitors of HRI, which were originally designed for the treatment of certain types of anemia [62], should be considered as an adjuvant therapy to cancer.

At first glance, the concept of mTORC1 activation as part of an anti-cancer strategy is counterintuitive to the pro-oncogenic role of mTORC1 in cancer. Pharmacological activators of mTORC1 were accordingly excluded for a pro-cancer potental. However, tuberous sclerosis patients, in which mTORC1 is somatically induced, develop tumors, mostly benign, over years and even decades, suggests that pharmacological promoters of mTORC1 should be safe. Moreover, the incidents of hematological cancers in tuberous sclerosis patients are rare, and not higher than in the general population [63], suggesting pharmacological activators of mTORC1 should not exacerbate MM on their own. Our data suggest that short exposures of tumors with an a priori low mTORC1 activity to mTORC1-activating drugs in combination with PIs should have a beneficial outcome.

How does mTORC1 activation predispose the mitochondria to develop stress? Clues on the mechanism may be derived by a better understanding of the mechanisms of HRI activation. HRI is activated by mitochondrial stress by an intriguing mechanism that was recently elucidated. The current understanding implicates the mitochondrial inner membrane protease OMA1 as the initiator of the response. Once activated, OMA1 cleaves DELE1 and releases a portion of it to the cytoplasm, where it binds HRI and meadiates its activation [44, 45]. In the absence of a structure of OMA1 and specific inhibitors, the molecular details of OMA1 activation are not known, despite being activated within minutes upon exposure of HEK293T cells to mitochondrial uncouplers [64]. We suggest that mTORC1 activation alters the mitochondrial membrane and/or mitochondrial proteostasis in a manner that facilitates OMA1 activation by stress conditions, perhaps related to the biophysical properties of the mitochondrial membranes. Regardless of the exact molecular mechanisms, a block of OMA1 activation and the availability of OMA1 inhibitors may provide a therapeutic alternative to HRI inhibitors.

Pharmacologically, from the drugs we tested, only PMA induced mTORC1 in a manner that resisted the suppression by PIs (Fig. 5C). The mechanism by which PMA promotes mTORC1 is unclear, but was suggested to depend on TSC2 [65] and phosphatidic acid synthesis [66]. PMA is used for promoting differentiation of monocytes to macrophages and for inducton of experimental inflammation especially in the skin [67]. As an agent that promotes differentiation, PMA was proposed to treat myeloid leukemias, efforts that did not proceed beyond phase 1 clinical trials, due to severe side effects [68]. We therefore did not pursue the combination of PMA and PIs in vivo, when given systemically. This does not exclude a targeted delivery of PMA to myeloma by virtue of ligand-coated liposomes or other drug delivery systems [69]. We reasoned that this should minimize the PMA-associated side effects and allow to re-evaluate PMA as an agent for myeloma, or other tumor types that can be efficiently targeted. However, liposomal encapsulated PMA was still toxic to mice prior to affecting mTOR activity in vivo (not shown). Developing direct activators of mTORC1 that target TSC are thus needed. The recently solved structure of the TSC2 in complex with Rheb [70], may provide information for a rational design of such TSC inhibitors. Based on the limited contacts between TSC2 and Rheb, it is conceivable that small molecules will be able to perturb the interaction of TSC2 and Rheb. This should be the best strategy to design mTORC1 activators, rather than targeting upstream regulators, such as AKT, AMPK or the use of amino acid analogs.

## Methods

**Cell culture**: HEK293T, Mel624, GL261, U87, and MIA PaCa-2 cells were cultured in high glucose DMEM (Sigma-Aldrich, D5796), RPMI8226 cells were cultured in DMEM/F-12 (Sigma-Aldrich, D6421), MM.1S cells were cultured in RPMI 1640 (Gibco, 21875034). Media was supplemented with 10% fetal bovine serum (Gibco, 12657029), 2 mM L-glutamine (Biological Industries, 03–020), 1% penicillin-streptomycin solution (Biological Industries, 03–031), and 1 mM sodium pyruvate (Biological Industries, 03–042). For amino acid starvation, DMEM/F-12 (USBiological, D9807–11) was used followed by supplementation with 10% dialyzed serum (Biological Industries, 04-011-1 A) and 25 mM glucose. Cells were maintained in humidified incubator with 5% CO_2_ and 37 °C.

**Chemical reagents**: ixazomib (Cayman, 18385), marizomib (Sigma-Aldrich, SML1916) oligomycin, FCCP and rotenone+antimycin A (Agilent, 103015–100) GCN-IN-1 (MCE, HY-100877), dorsomorphin (Cayman, 11967), MHY1485 (Sigma-Aldrich, SML0810), PMA (Sigma-Aldrich, P1585), rapamycin (LC Laboratories, R-5000), vemurafenib (Cayman, 10618) celecoxib (Sigma-Aldrich, SML3031).

**TSC2/HIF1β mRNA correlation**: The correlation between mRNA expression level between mTOR and HIF1β in CD138 + bone marrow plasma cells from healthy subjects (*n* = 22) and newly diagnosed MM patients (*n* = 559). Data were obtained from Gene Expression Omnibus database available online (GSE2658 and GSE5900) [71].

### Analysis of gene expression in Myeloma samples

Gene expression profiling of bone marrow PCs was performed as described previously [72]. Briefly, RNA extraction was performed using the RNeasy kit (Qiagen, Hilden, Germany), the SV-total RNA extraction kit (Promega, Mannheim, Germany) and Trizol (Invitrogen, Karlsruhe, Germany). Labeled cRNA was generated using the small sample labeling protocol vII (Affymetrix, Santa Clara, CA, USA), and hybridized to U133 2.0 plus arrays, according to the manufacturer’s instructions. Expression data were pre-processed using GC-RMA normalization and the Affymetrix U133 Version 2.0 plus array custom CDF (v25) mapping to Entrez genes (http://brainarray.mhri.med.umich.edu/Brainarray/Database/CustomCDF/). Absolute gene expression levels are displayed as log2-transformed values. The expression data are deposited in ArrayExpress under the accession numbers E-MTAB-81 and E-GEOD-2658. For an in-depth sample overview, refer to [73].

**Hypoxia induction:** hypoxia incubator chamber (Stemcell technologies, cat number 27310) was sterilized with 70% ethanol and humidified with sterile water. Plated cells were placed inside the chamber, thereafter, chamber was sealed and purged with 1% O_2_ for 15 min. Valves were tightly closed and chamber was placed in 37 °C incubator.

**Mitochondrial respiration measurements:** oxygen consumption rate (OCR) measurements were performed in Seahorse XFe96 Analyzer (Agilent) by using Mito Stress test kit (Agilent, 103015–100) according to the manufacturer’s instructions. Cells were seeded at a density of 3 × 10^4^ cells per well in a Seahorse XF RPMI assay medium into a 96-well plate. Oligomycin and FCCP were added to a final concentration of 2 μM, rotenone and antimycin A were added to a final concentration of 0.5 μM. OCR parameters were calculated as detailed in Table 1.

**Table 1:** Equations for calculations of mitochondrial activity.

Basal respiration	(Late rate measurement before first injection) – (non-mitochondrial respiration rate)
Proton leak	(Minimum rate measurement after oligomycin injection) – (non-mitochondrial respiration rate)
Maximal respiration	(Maximum rate measurement after FCCP injection) – (non-mitochondrial respiration rate)
Spare respiratory capacity	(Maximal respiration) – (basal respiration)
Non-mitochondrial oxygen consumption	Minimum rate measurement after rotenone/antimycin A injection
ATP production	(Late rate measurement before oligomycin injection) – (minimum rate measurement after oligomycin injection)

**Transcriptome sequencing:** total RNA was isolated using BioTri reagent (Bio-lab, 959758027100), 1 ml of the reagent was used to lyse 5–10 × 10^6^ cells. After phase separation, 0.2 ml of chloroform was added and samples were vortexed for 15 s and incubated for 5 min at RT, then centrifuged (12,000 × *g*, 4 °C for 15 min). The upper aqueous phase was transferred to a new tube, mixed, and incubated with 0.5 ml isopropanol for 10 min, then samples were centrifuged (12,000 × *g*, 4 ˚C for 10 min). Produced pellet was washed with 1 ml ethanol (75%), then centrifuged (12,000 × *g*, 4 °C for 5 min). Produced pellet was dried and resuspended with 20–50 μl of ultra-pure water. RNA quality was assessed using RNA ScreenTape (Agilent, 5067–5576) on Agilent 4200 TapeStation. RINe score of 10 was confirmed for all samples. Transcriptome sequencing libraries for mRNA were prepared from 1 µg of RNA using KAPA Stranded mRNA-Seq Kit (Kapa biosystems, KK8421) and according to the manufacturer’s instructions. The STAR software [74] was used for alignment of the generated transcriptome, GRCh37 was used as a reference sequence for transcriptome mapping. For quantitative analysis and differential gene expression, HTSeq [75] and Deseq2 [76] were used, respectively. *P*-value < 0.05 was defined and other parameters were kept default.

**Generation of knock out cells using CRISPR-Cas9:** sgRNAs were designed by using genetic perturbation platform (GPP) sgRNA designer of Broad institute [77]. sgRNAs were cloned into lentiCRISPR v2 (addgene #52961). sgRNAs sequences that were used are as following:

Human TSC2: 5′-CAGAGGGTAACGATGAACAG-3′.

Human NPRL2: 5′-GGTTGAAGAGGAGAGCATTG-3′.

Human ATG7: 5′-CGGCTCCAGAAAATATTCCC-3′.

Human HRI: 5′-ATAGTCGAGAGAAACAAGCG-3′.

Human ATF4: 5′-TCTCTTAGATGATTACCTGG-3′.

Mouse TSC2: 5′-CACAGGGTGATAATGAACAG-3′.

Mouse ATG7: 5′-GAGAGCATCCCTCTAATCCG-3′.

For lentiviruses production, a mix of sgRNA-lentiCRISPR v2, pCMV-dR8.2 dvpr (addgene #8455) and pCMV-VSV-G (addgene #8454) was transfected to HEK293T cells in a respective ratio of 3:2:1. PEI (Sigma-Aldrich, 764604) was used as a transfection agent. Following 48 h, lentivirus containing media was collected, mixed with polybrene (Millipore, TR-1003-G) and added to the cells for 24 h. Transduced cells were selected with 0.5 μg/ml puromycin (Sigma-Aldrich, P9620) for 72 h, then cells were subcloned in a 96-well plate. Single-cell clones were expanded and screened by immunoblotting of the relevant protein.

**Immunoblotting:** Cells were harvested by centrifugation (4 °C, 4000 × *g* for 5 min) and washed with ice cold PBS. Cell pellets were lysed with RIPA buffer supplemented with protease inhibitors (Bimake, b14001) and phosphatase inhibitors (Bimake, b15001). Following agitation (4 °C, 7xrpm for 10 min), lysates were centrifuged (4 °C, 16,000 × *g* for 15 min), supernatant was separated by micropipette, quantified, and mixed with sample buffer thereafter. Purified lysate was denaturized by boiling (95 °C for 5 min). Samples were loaded on SDS-PAGE and resolved by electrophoresis (120 V) and then transferred to PVDF membrane (4 °C, 100 V, for 1.5 h). Thereafter, membrane was blocked with 5% skim milk dissolved in TBST (at RT for 1 h), washed (3 times, 5 min each), then incubated with primary antibody (at 4 °C for 16–24 h), washed then incubated with anti-mouse or anti-rabbit horseradish peroxidase (HRP)-conjugated secondary antibody (at RT for 1 h), washed then detected by chemiluminescence using Bio-Rad ChemiDoc™ XR. Immobilon^®^ Crescendo (Millipore, WBLUR0500) was used as chemiluminescent substrate for HRP. Antibodies were used according to the manufacturer’s instruction and listed here: anti-PS6 (CST #5364) anti-S6 (CST #2217), anti-P4EBP1 (CST #9459), anti-4EBP1 (CST #9644), anti-S6K1 (CST #9202), anti-PS6K1 (CST #9205), anti-p97 (was kindly provided by Dr. Hidde Ploegh, Boston Children’s Hospital), anti-ubiquitin (kindly provided Dr. Ariel Stanhil, The Open university of Israel), anti-ATG7 (CST #8558), anti-LC3B (CST #2775), anti-p62 (abcam ab91526), anti-actin (abcam, ab49900), anti-ATF4 (CST #11815), anti-PERK (CST #5683), anti-TSC2 (CST #4308), anti-NPRL2 (CST #37344), anti-HRI (MBS, MBS2538144), anti-PAMPKα (abcam, ab194920), anti-AMPKα (CST #2532), anti-AKT (CST #9272) anti-PAKT (CST #4056), anti-cleaved caspase-3(CST # #9664), goat anti-mouse and goat anti-rabbit HRP-conjugated secondary antibodies were purchased from Jackson Laboratories.

**Flow cytometry:** cells were harvested, washed with PBS and filtered through a 100 μm strainer. 10^4^ cells per sample were analyzed by using Cytoflex flow cytometer (Beckman Coulter). Data processing was performed by using CytExpert software (Beckman Coulter).

**Cell viability measurements:** cells were harvested, washed with PBS and filtered through a 100 μm strainer. Propidium iodide (Sigma-Aldrich, P4170) was added to a final concentration of 1 μg/mL and cells were analyzed by flow cytometry. Viable cells were measured by gating on propidium iodide unstained cells with respect to their scattering properties.

**MTT assay:** MTT (Calbiochem, 475989) was added to the cells to a final concentration of 0.5 mg/ml in a 96-well plate, then cells were incubated in humidified incubator with 5% CO_2_ and 37 °C for 4 h. Thereafter, media was removed and 200 μl of DMSO was added to each well. Absorbance was measured at wavelength of 595 nm using plate reader.

**Mitochondrial membrane potential measurement:** JC-1 (abcam, ab113850) was added to the cells to a final concentration of 1 μM for 30 min. Thereafter, cells were harvested by centrifugation, washed with PBS and filtered through a 100 μm strainer. Then, cells were analyzed by flow cytometry. Compensation was performed uniformly to all samples by subtracting green channel (FITC) from red channel (PE).

**In vivo growth of MM.1S xenograft**: wt, NPRL2 KO and TSC2 KO MM.1 S cells were inoculated subcutaneously into *n* = 4 NCG mice (male, 8-week-old, Charles River). Four weeks later, tumors were resected and weighted. Each mouse was inoculated with 2 × 10^6^ cells of each cell type, hence each mouse served as a control to themselves, and tumor growth was normalized to their own WT.

**Immunofluorescence and microscopy**: bone marrow biopsies that were taken from primary MM patients were washed three times with PBS and fixed with 4% PFA/PBS (at -RT for 20 min). After fixation and washing, cells were permeabilized with 0.25% Triton X-100 (at RT for 10 min), washed with PBS and blocked by 5% FCS/PBS for 1 h. Diluted fluorescent P-S6 and Total-S6 antibodies (CST #9468, R&D #IC5436G) were added (1:100 each) and incubated overnight at 4 °C. Then, cells were washed and mounted with coverslip glass. Slides were imaged by using Olympus FV10i confocal microscope (OLYMPUS). Brightness and contrast were adjusted equally to all images. Quantification of P-S6/S6 mean fluorescent intensity (MFI) was measured by ImageJ software.

## Supplementary information

Supplemental figures

Uncropped immunoblots

## Data Availability

All data generated and/or analyzed in this article are available from the corresponding author upon reasonable request.
