# Supplementary material for: An mTORC1 to HRI signaling axis promotes cytotoxicity of proteasome inhibitors in multiple myeloma
Source: Cell Death Dis. 2022 Nov 18;13(11):969. doi: 10.1038/s41419-022-05421-4 (PMC9674573; doi:10.1038/s41419-022-05421-4)
Supplement: Supplementary file 1 — Supplemental figures [file 41419_2022_5421_MOESM1_ESM.pdf]

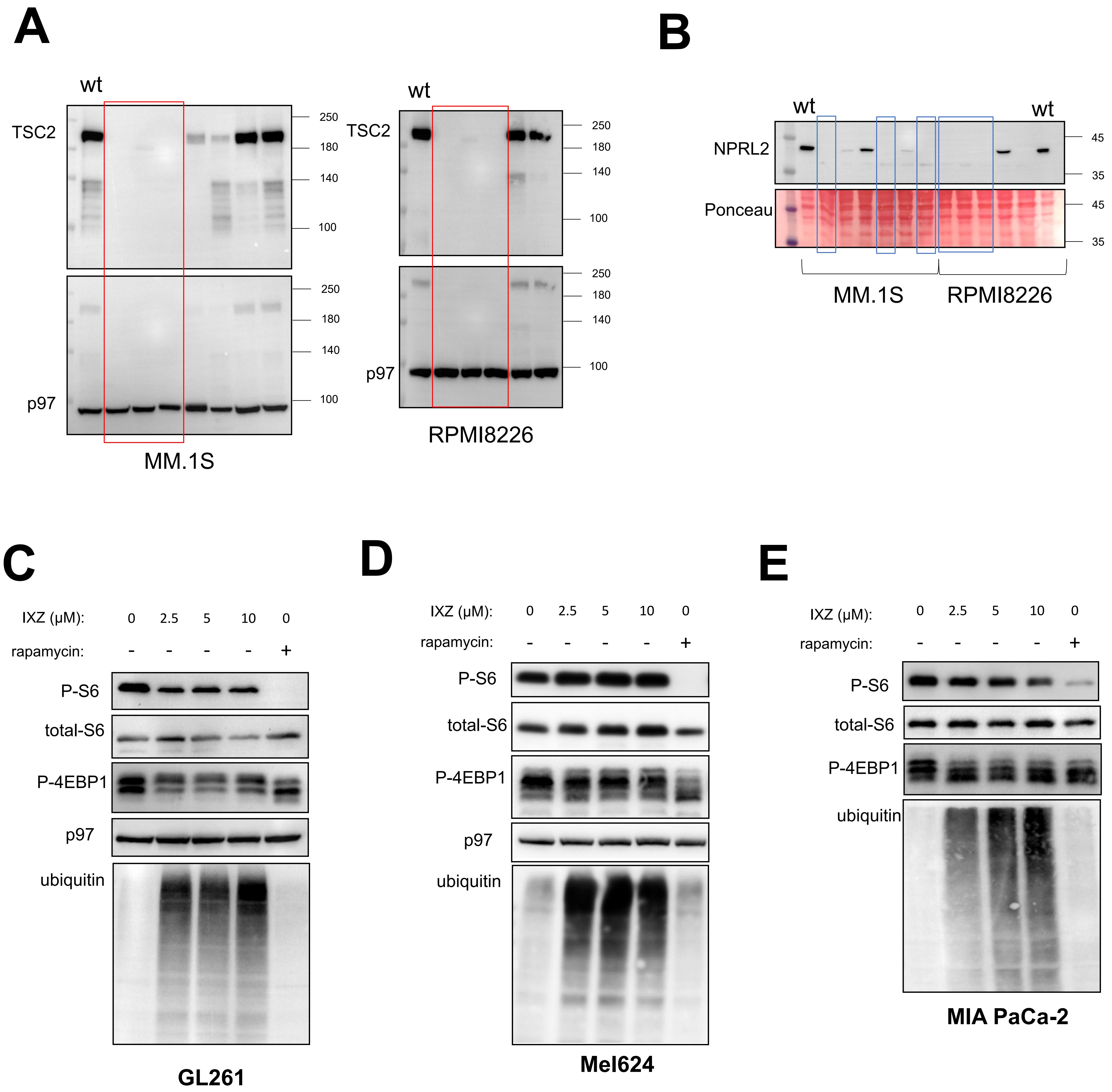

Figure S1: Generation of TSC2 KO (A) and NPRL2 KO (B) in MM.1S and RPMI8226 cells. p97 immunoblot and ponceau staining were used to confirm an equal protein loading in each lane. (C-E) A dose-response analysis of mTORC1 activity following IXZ treatment for 12h was examined by immunoblotting against its downstream phosphorylated effectors, S6 and 4E-BP1. Rapamycin [50 nM] treatment was performed in parallel as a positive control for mTORC1 suppression. Ubiquitin immunoblot was used to confirm proteasomal inhibition by IXZ treatment. Shown are a typical immunoblots out of three independent experiments.

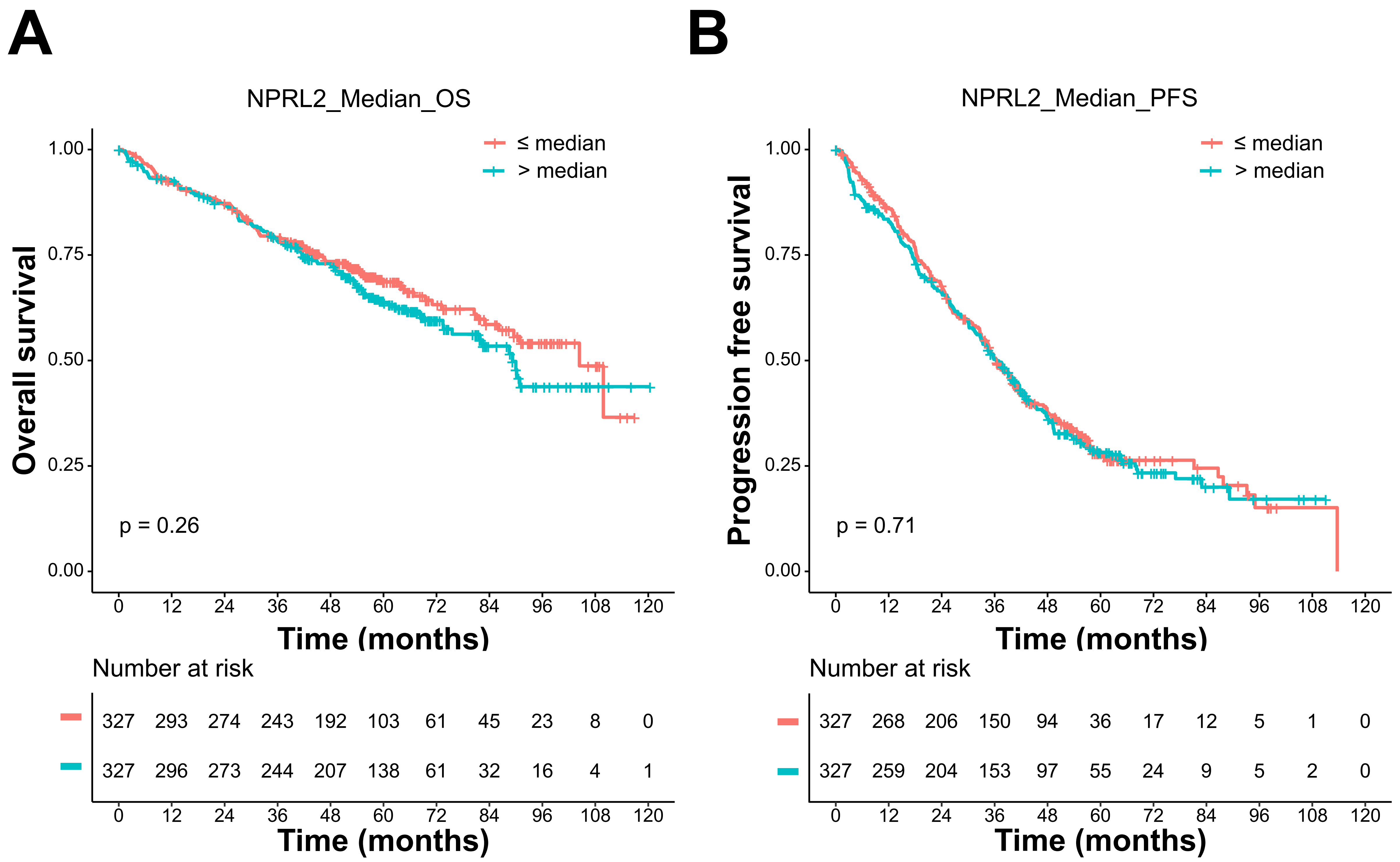

Figure S2: NPRL2 expression does not affect the overall survival (OS) and progression free survival (PFS) of MM patients. Shown are Kaplan-Meier plots for the OS (A) and PFS (B) of MM patients with differential expression of NPRL2 from GMMG HD4 and MM5 trials.

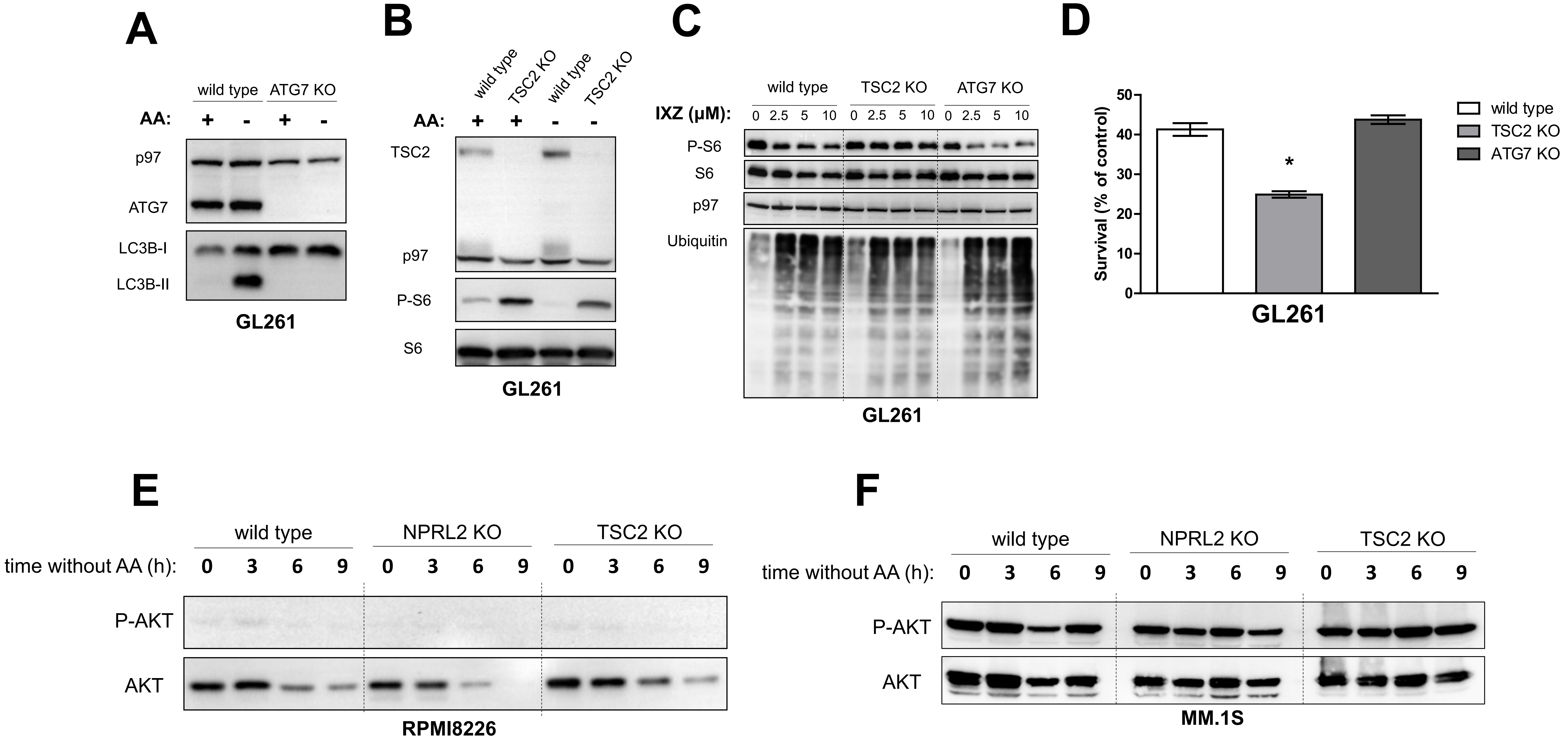

Figure S3: Generation of ATG7 KO (A) and TSC2 KO (B) GL261 cells. Shown are typical immunoblots against downstream effectors of autophagy and mTORC1 in AA replete and deplete conditions as indicated. Complete deletion of ATG7 and TSC2 was achieved, and readout of these syngeneic cells was observed as expected. LC3B-I to LC3B-II conversion was completely abolished by ATG7 deletion in both AA full and free media (A). S6 phosphorylation was upregulated in TSC2 KO in both AA full and free media compared to wt cells (B). (C) GL261 cells were treated with IXZ for 12h. Then, mTORC1 activity was assessed as described in Fig 1. (D) GL261 cells were treated with IXZ [2.5 µM] for 48h. Shown is average relative viability of three independent experiment ± S.E.M., *p<0.05 of unpaired two-tailed student’s t-test between wt and TSC2 KO. (E, F) AKT activity was assessed by immunoblotting against its phosphorylated T-308 and total form in different time points following AA starvation.

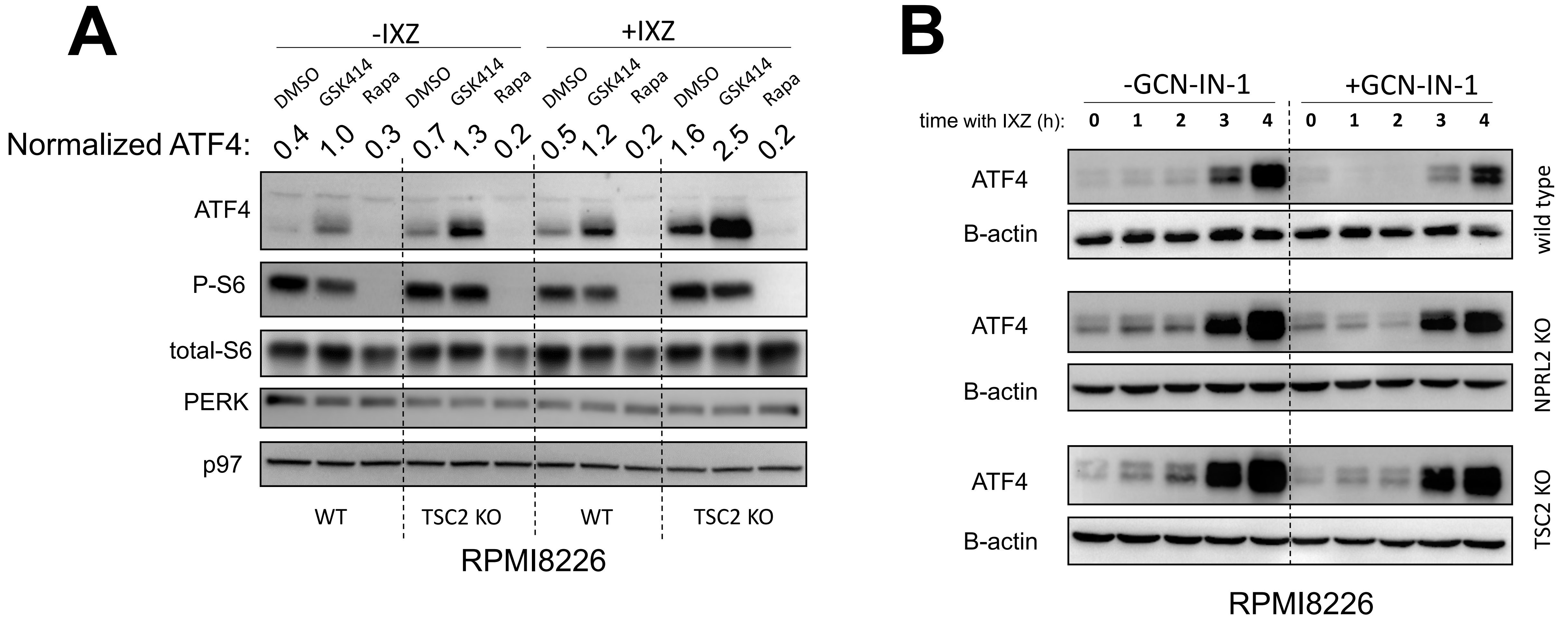

Figure S4: ATF4 induction by PI is not mediated through PERK or GCN2. (A) cells were pretreated with the PERK inhibitor, GSK414 [1 µM], or mTORC1 inhibitor, rapamycin [50 nM], or DMSO as a vehicle control. Each treated cell population was either treated with IXZ [32 nM] or left untreated for 4h. ATF4 induction was observed by immunoblotting. Phospho and total S6 immunoblots were used to confirm mTORC1 suppression by rapamycin. PERK immunoblot was used to assesses its activity. Normalized ATF4 was calculated by dividing band intensity of ATF4 immunoblot to its corresponding p97 immunoblot. (B) ATF4 induction was monitored following a time course treatment of IXZ [32 nM] with or without GCN2 inhibitor, GCN-IN-1 [10 µM], as indicated.

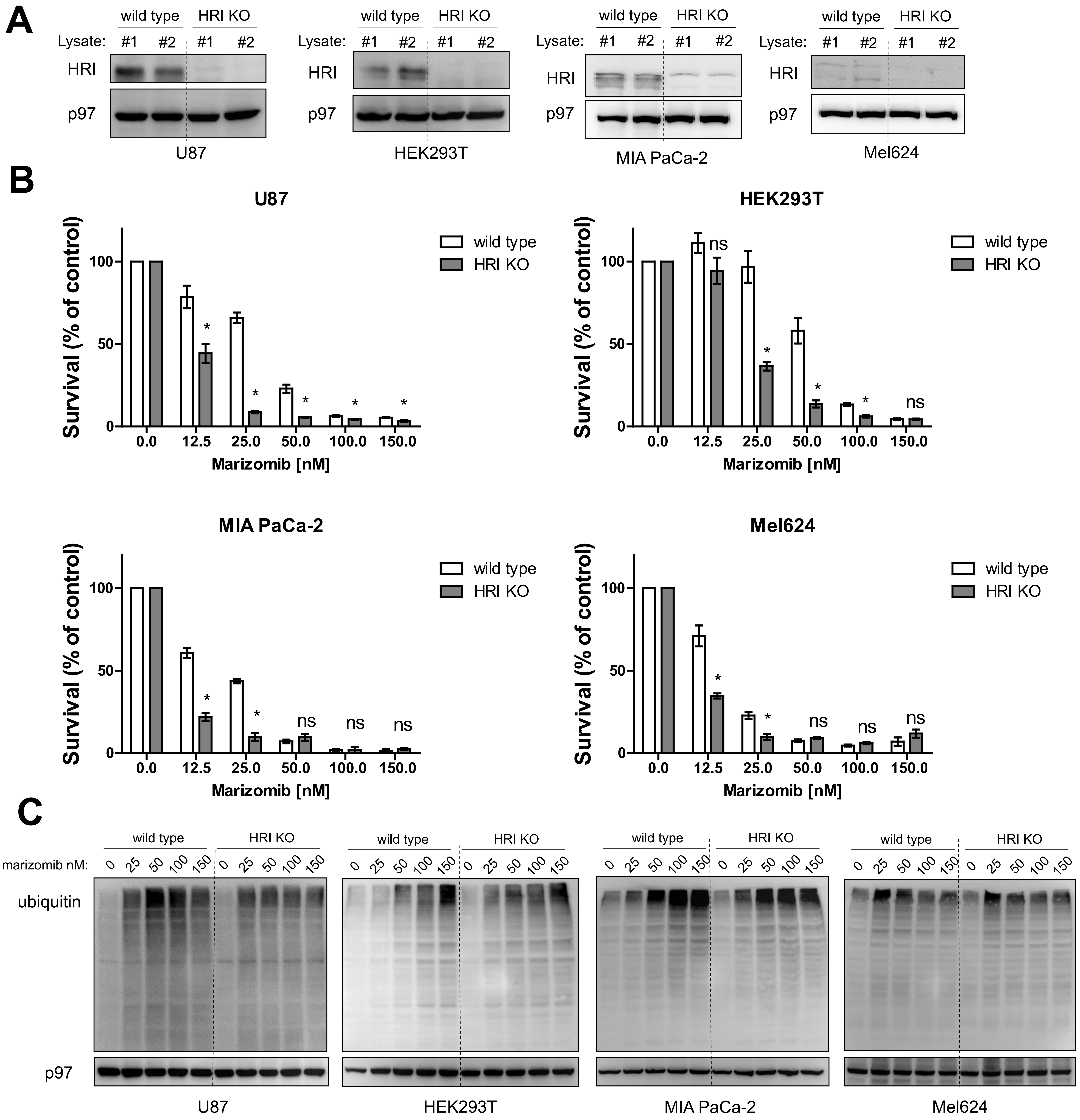

Figure S5: HRI deletion over-sensitizes cancerous cells to PI treatment. (A) Generation of HRI KO cells. Shown are representative immunoblots for wt HRI and KO HRI cells using two independent lysate preparations. (B) The indicated cells were treated with MRZ by a dose-escalating manner for 120h. Shown are the average relative viability of five replicates ± S.E.M., *$p<0.05$ of unpaired two-tailed student's t-test. ns, not significant. Viability was assessed by MTT assay. (C) Shown are immunoblots of ubiquitylated proteins following the treatment of MRZ as indicated for 24h. MRZ, marizomib.

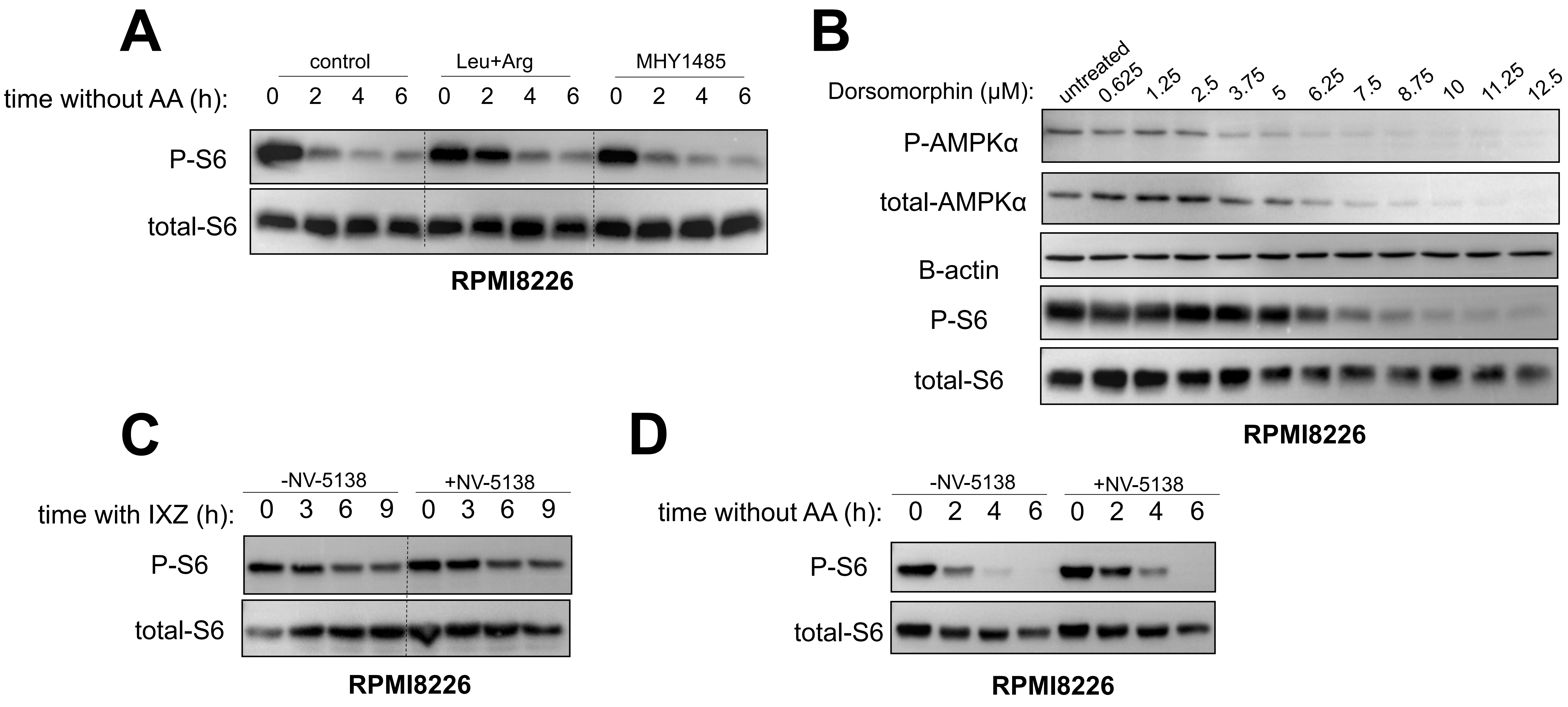

Figure S6: (A) A time-response analysis of mTORC1 activity following MHY1485 was assessed by immunoblotting against P-S6 and total-S6 in AA free media. An additional treatment with Leu+Arg was performed in parallel for comparison. (B) A dose-response analysis of mTORC1 activity following an overnight treatment with dorsomorphin was assessed by immunoblotting against P-S6 and total-S6. Inhibition of AMPK activity was confirmed by immunoblotting against P-AMPK and total-AMPK. B-actin immunoblot was performed to confirm an equal protein loading. (C) A time-response analysis of mTORC1 activity following IXZ treatment (C) or AA starvation (D) alone or in combination with NV-5138 [100 µM], as indicated.

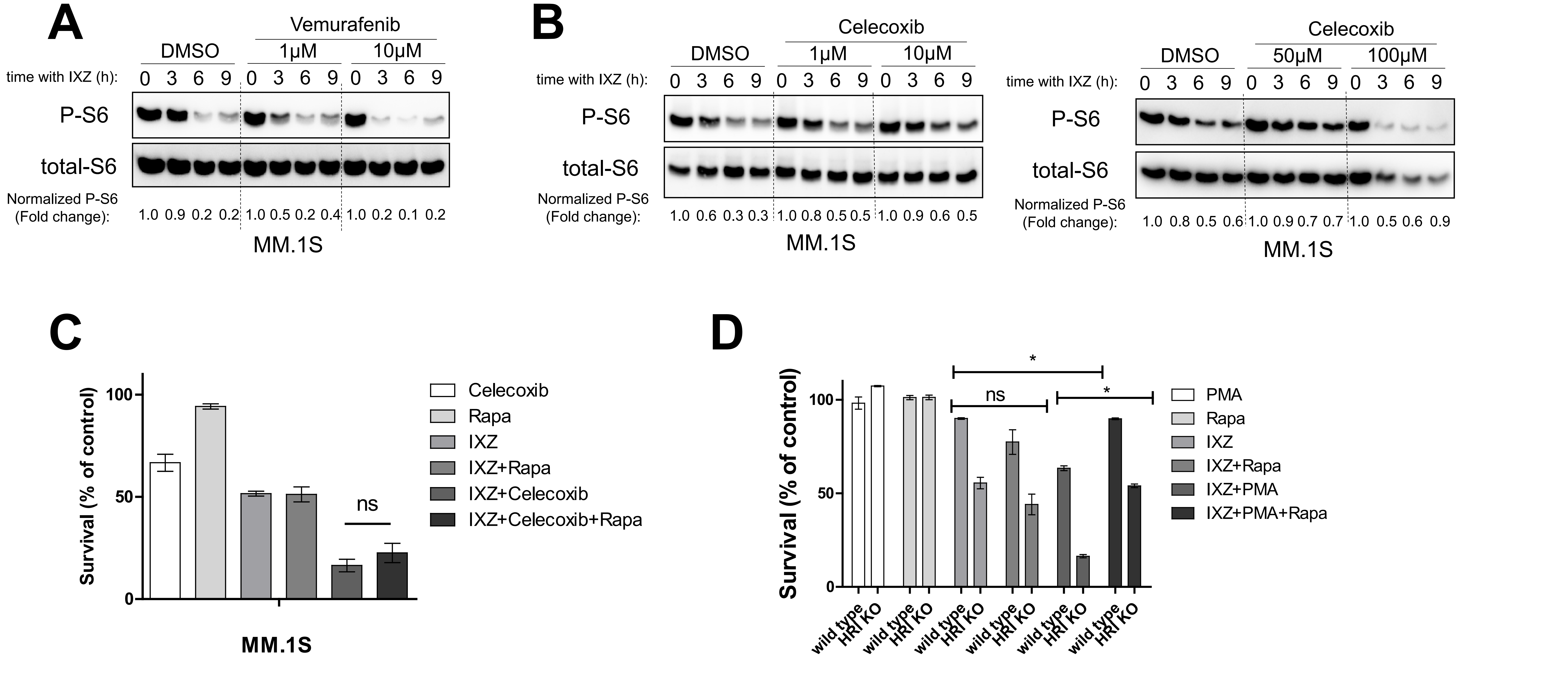

Figure S7: (A) analysis of mTORC1 activity was assessed following IXZ [32 nM] alone or in combination with vemurafenib (A) or celecoxib (B) at the indicated concentrations. (C) Shown is average relative viability of three technical measurements ± S.E.M. cells were treated with [IXZ 20 nM] [Rapa 50 nM] [Celecoxib 50 μM] alone or with combinations as indicated for 48h. ns, not significant. (D) Shown is average relative viability of two technical measurements ± S.E.M. cells were treated with [IXZ 10 nM] [Rapa 50 nM] [PMA 125 nM] alone or with combinations as indicated for 48h. ns, not significant. *p<0.05 of unpaired two-tailed student's t-test.

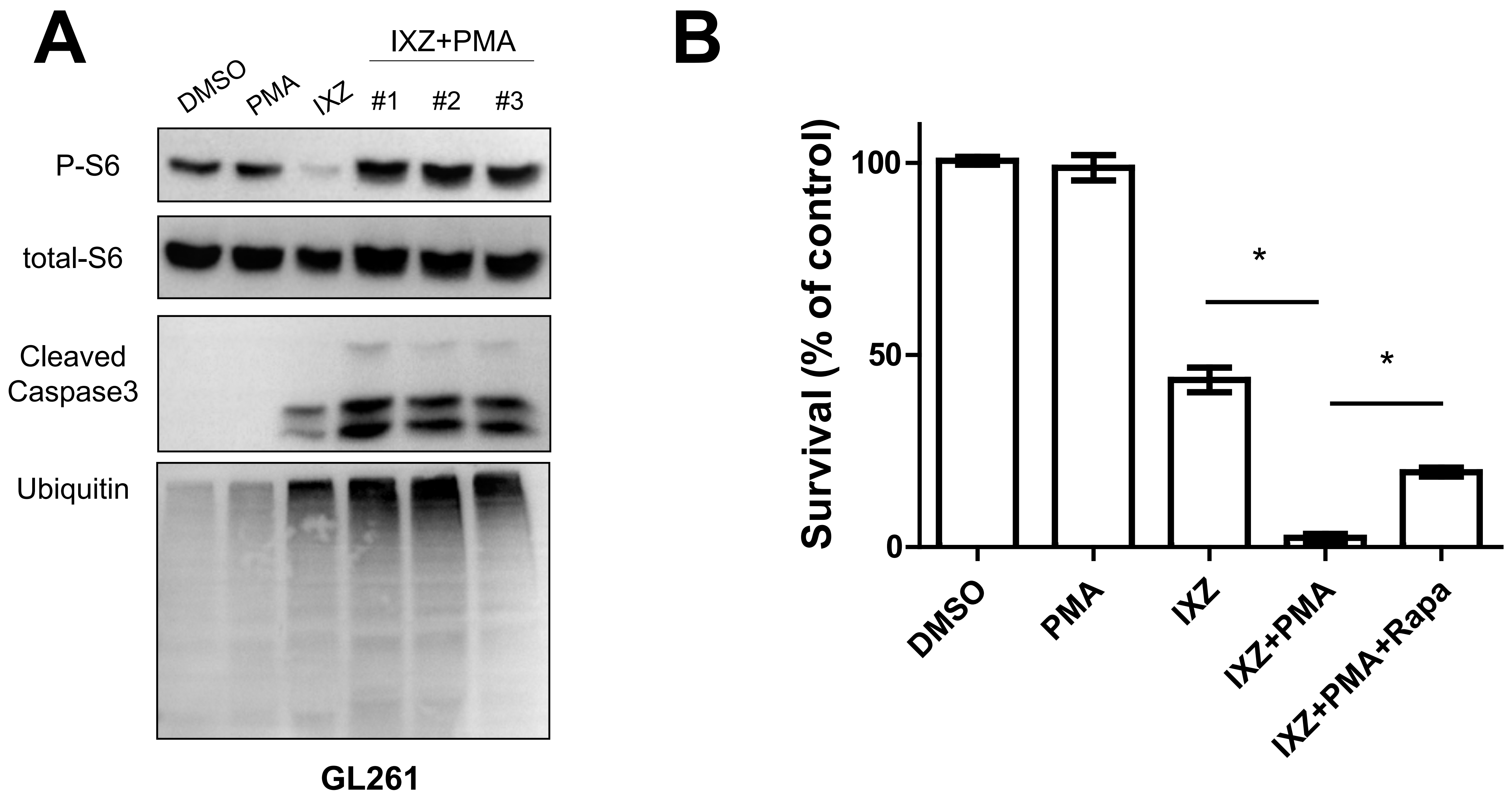

Figure S8: PMA sensitizes glioblastoma to IXZ**.** (A) analysis of mTORC1 activity was assessed following overnight treatment with PMA [125 nM], IXZ [2.5 μM] alone or in combination as indicated. Three technical repetitions are shown for the combined treatment. Immunoblot against cleaved caspase3 was used to assess apoptosis. Ubiquitin immunoblot was used to confirm proteasomal inhibition by IXZ treatment. (B) Shown is the relative average viability GL261 cells following treatment with the indicated drugs for 48h, concentration was used as follows: PMA [125 nM], IXZ [2.5 μM], rapamycin [50 nM]. Error bars represent ± S.E.M. of three independent experiments, *$p<0.05$ of unpaired two-tailed student's t-test.

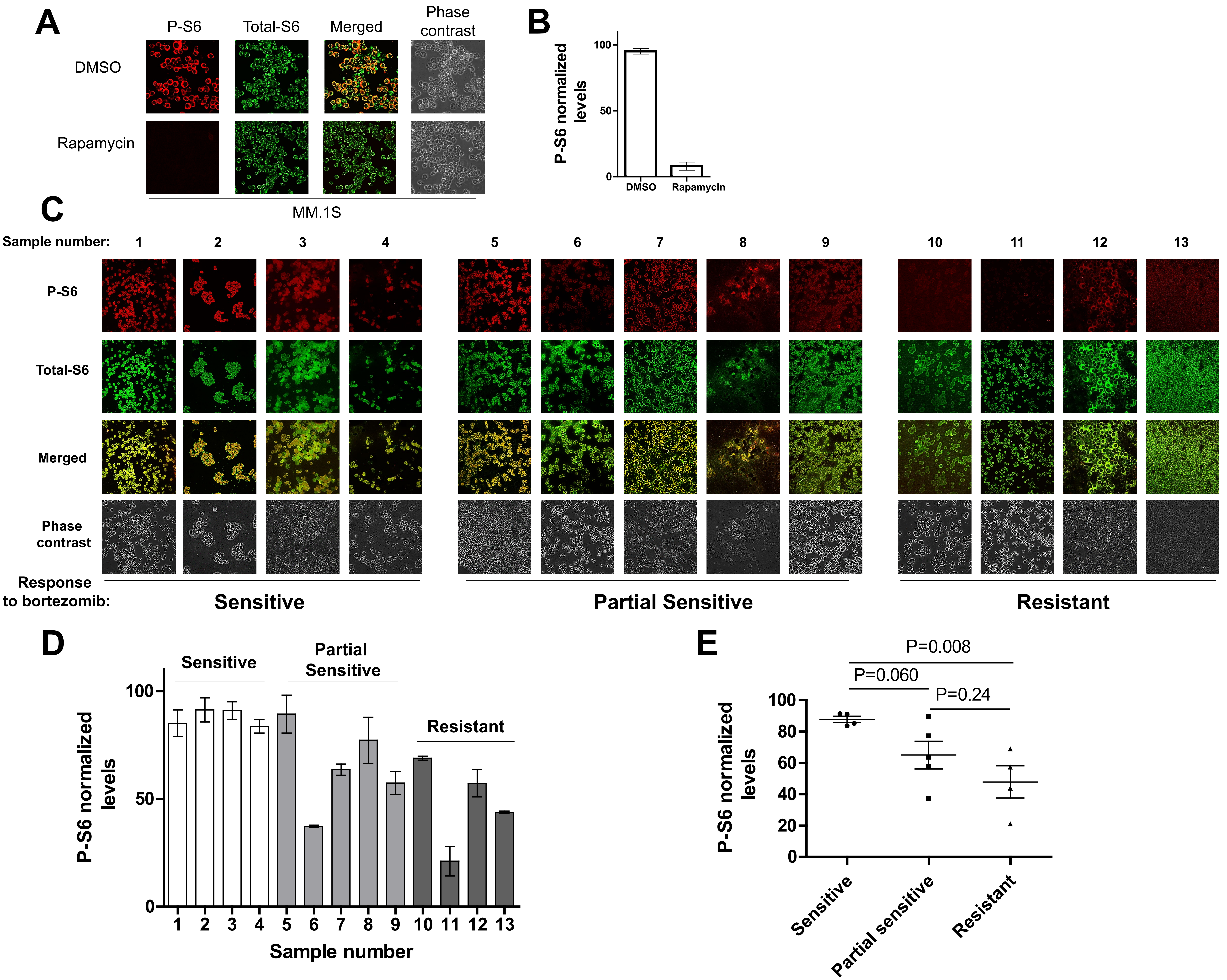

Figure S9: mTORC1 activity at the time of diagnosis is correlated with sensitivity to bortezomib. (A) MM.1S cells were treated with either DMSO or rapamycin [50 nM] for 16h. Shown are typical images of P-S6 (red) and total-S6 (green). (B) Quantification of normalized P-S6 shown in (A). (C) P-S6 (red) and total-S6 (green) in bone marrow smears taken from newly diagnosed MM patients were imaged and correlated with development of resistance to Bz. Shown is the result of 13 patients categorized to sensitive, partial sensitive and resistant. (D) Quantification of normalized P-S6 shown in (C). (E) Shown is grouped dot plot of (D). p-value was calculated by unpaired two-tailed student's t-test. Column graphs represent the average of two measurements and error bars represent ± S.E.M. of two images from same slide.
