# Supplementary material for: An mTORC1 to HRI signaling axis promotes cytotoxicity of proteasome inhibitors in multiple myeloma
Source: Cell Death Dis. 2022 Nov 18;13(11):969. doi: 10.1038/s41419-022-05421-4 (PMC9674573; doi:10.1038/s41419-022-05421-4)

Figure 1E

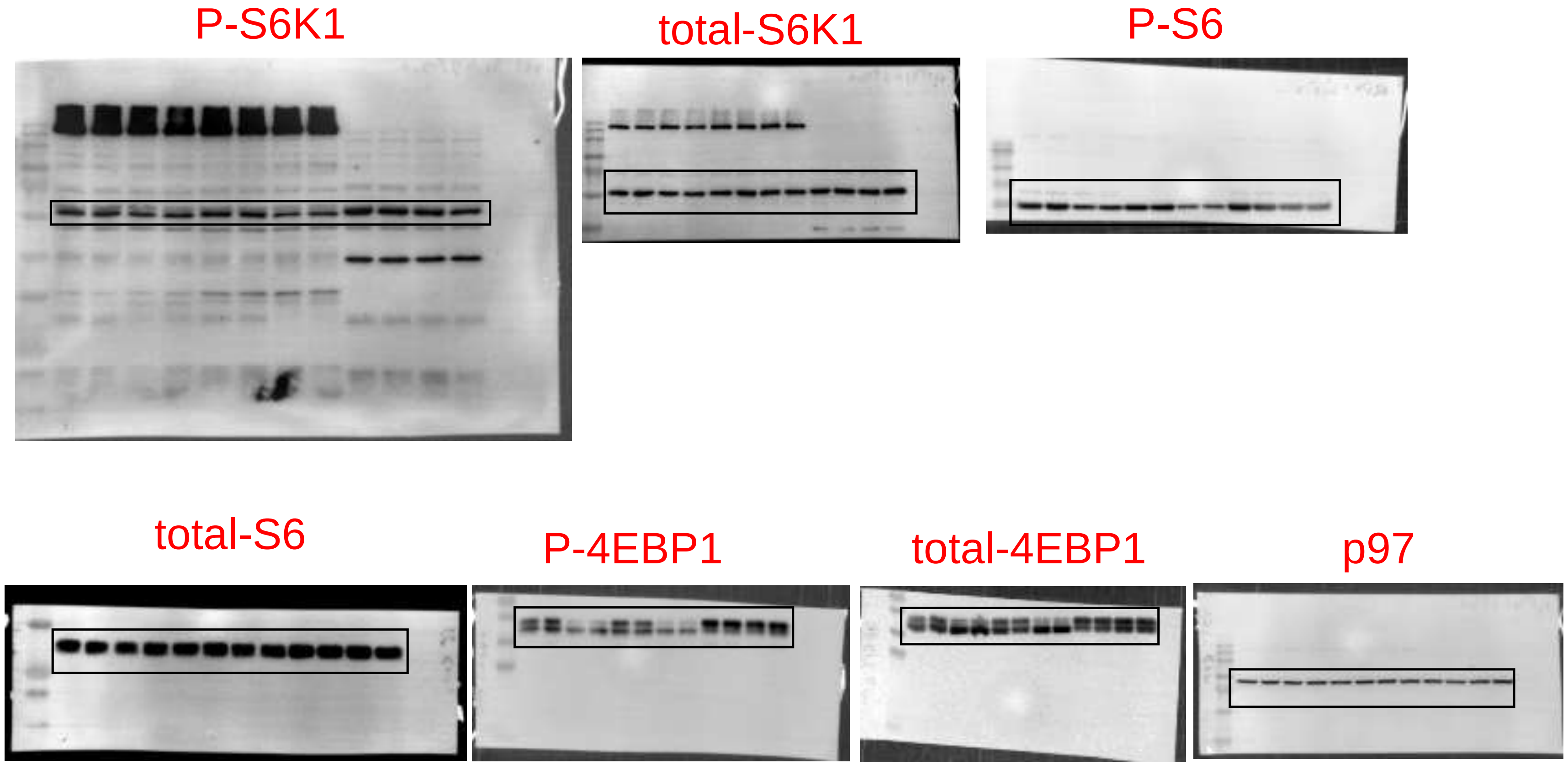

Figure 1F

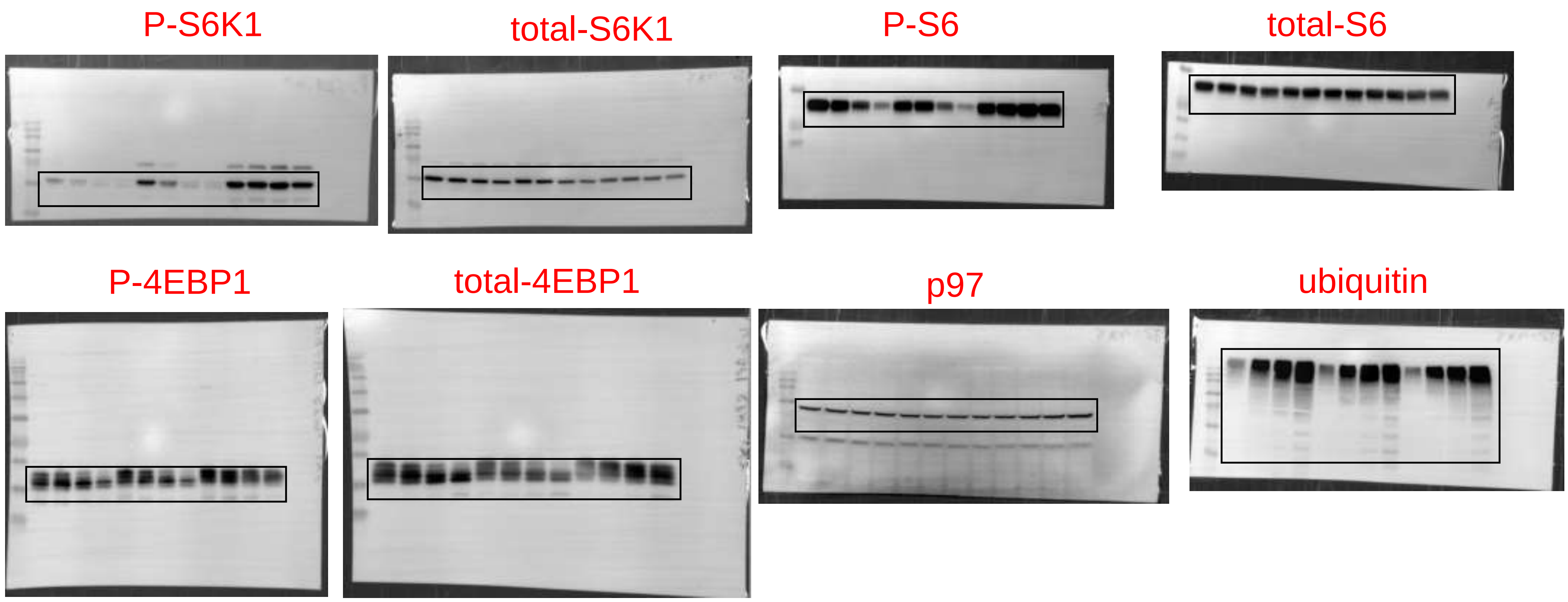
P-S6K1
total-S6K1
P-S6
total-S6
P-4EBP1
total-4EBP1
p97
ubiquitin

Figure 1G

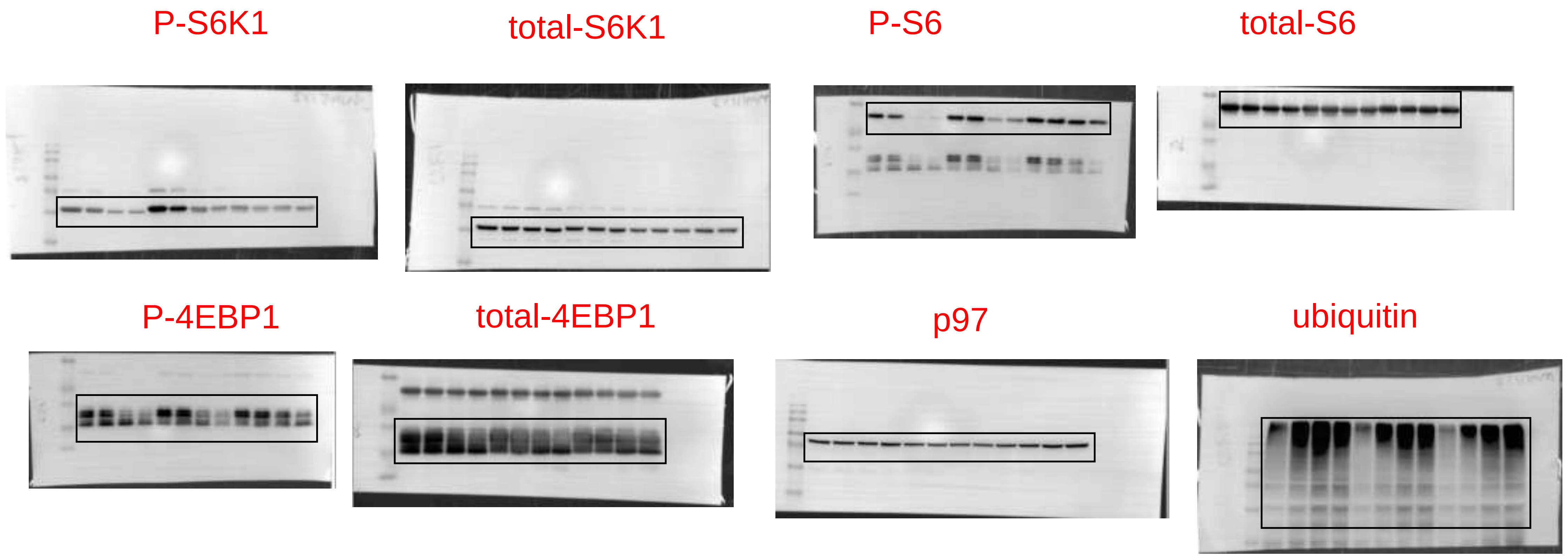
P-S6K1
total-S6K1
P-S6
total-S6
P-4EBP1
total-4EBP1
p97
ubiquitin

Figure S1A

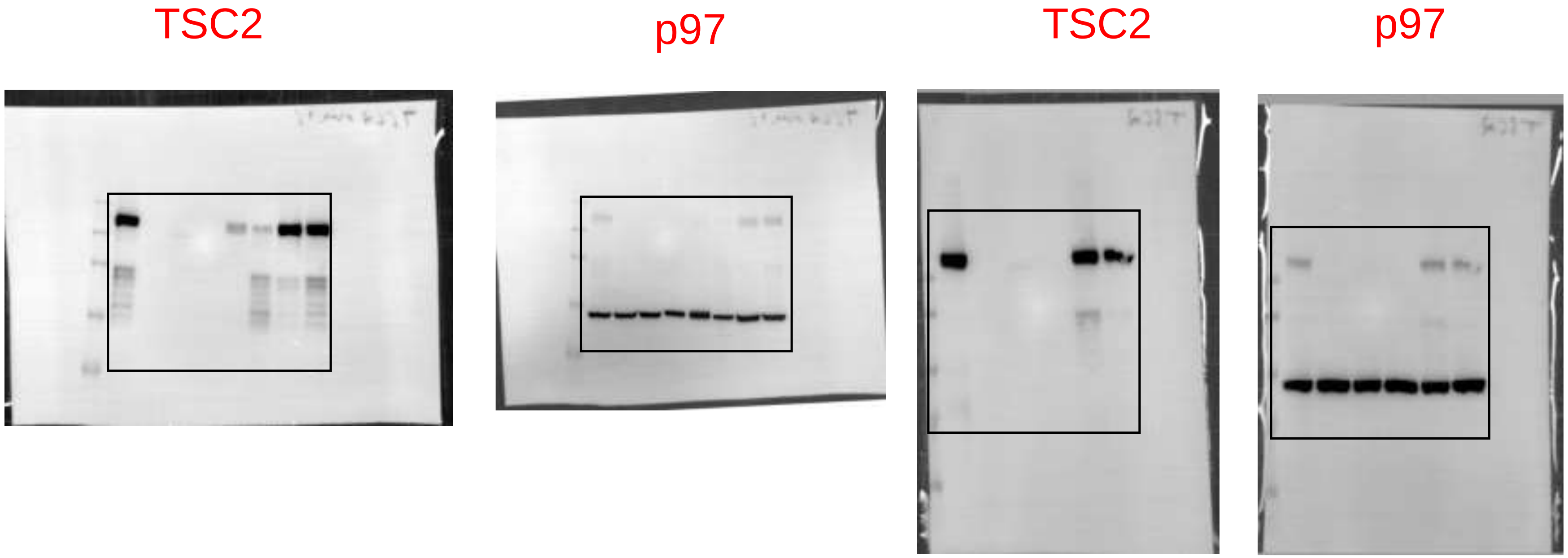

Figure S1B

NPRL2

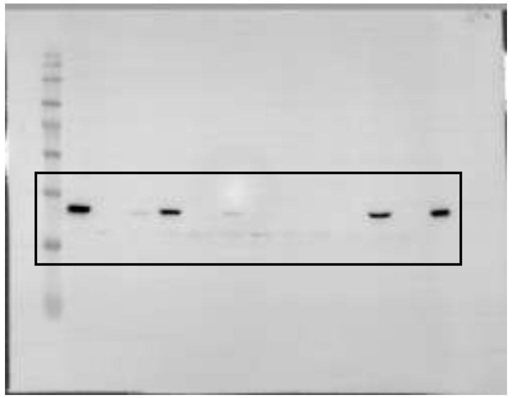

Ponceau

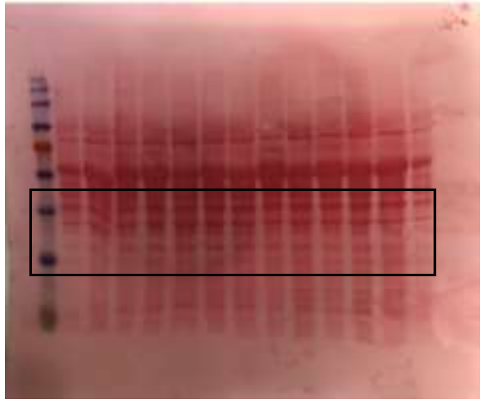

Figure S1C

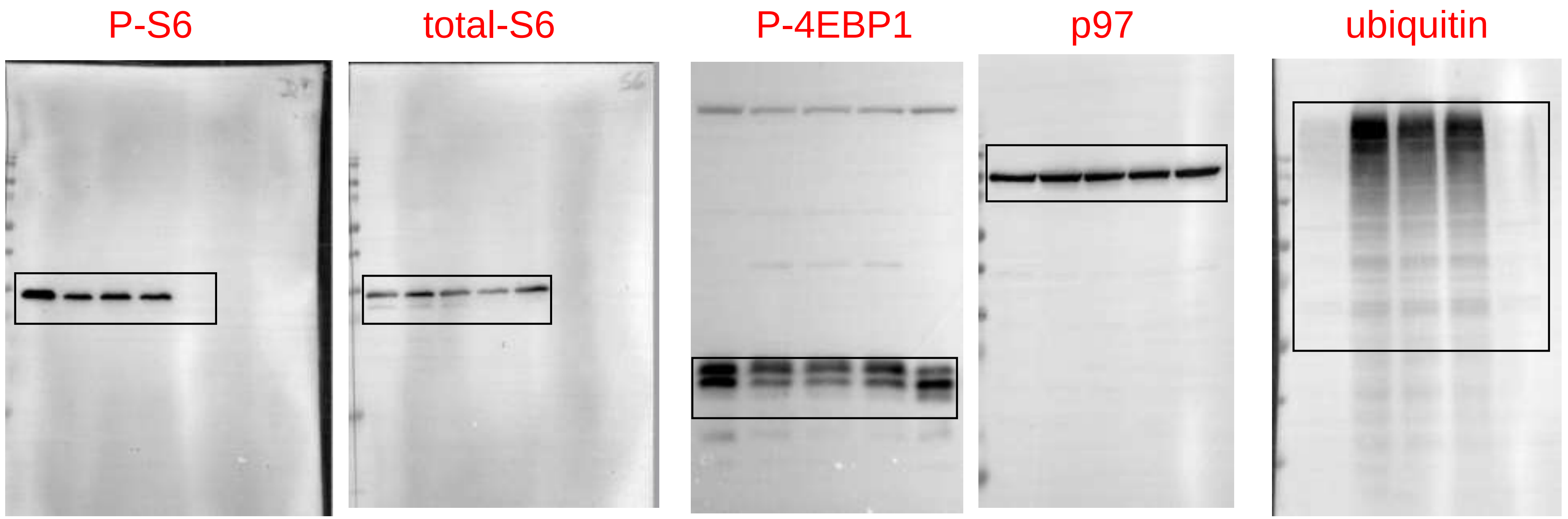

Figure S1D

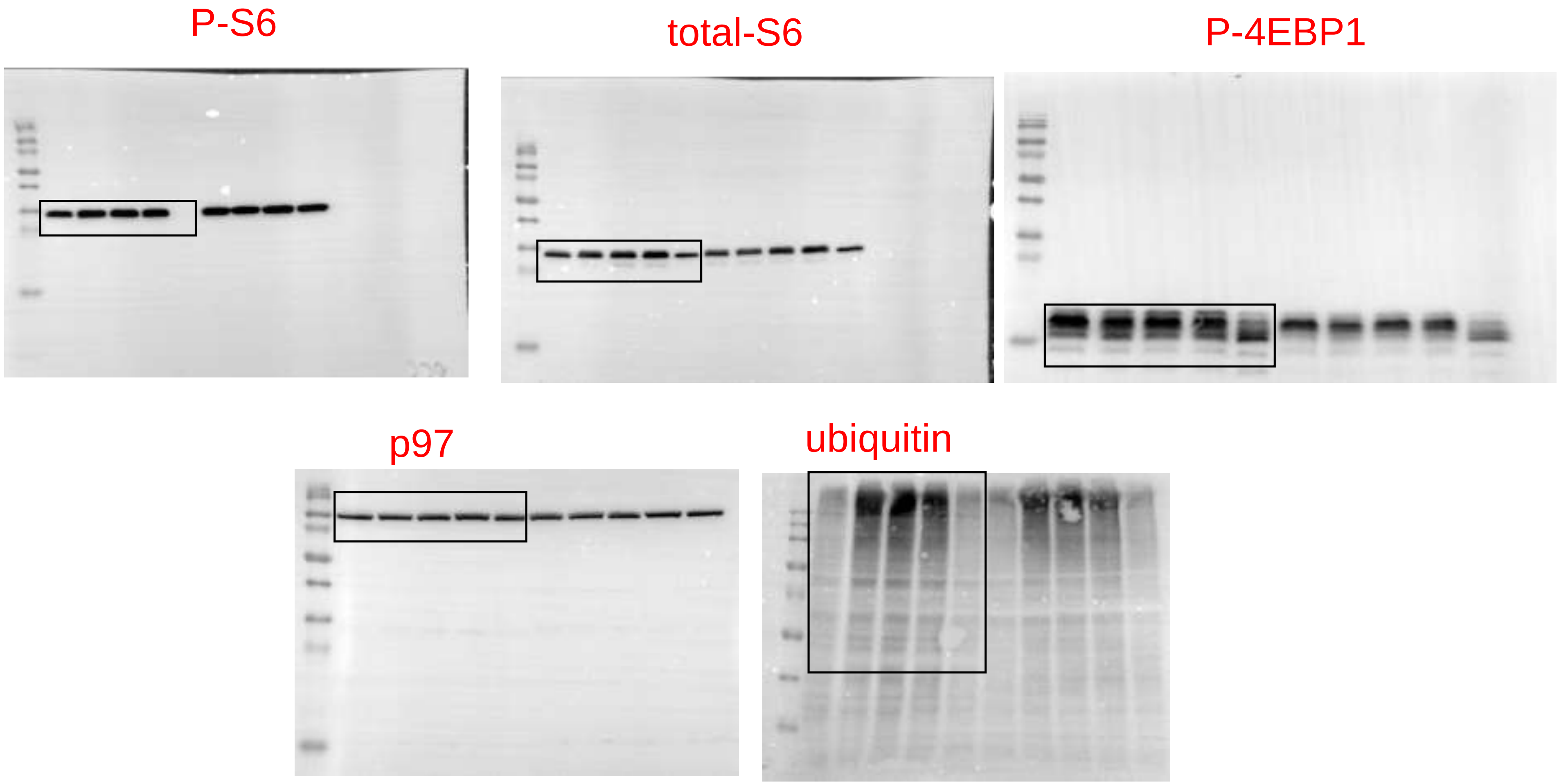

Figure S1E

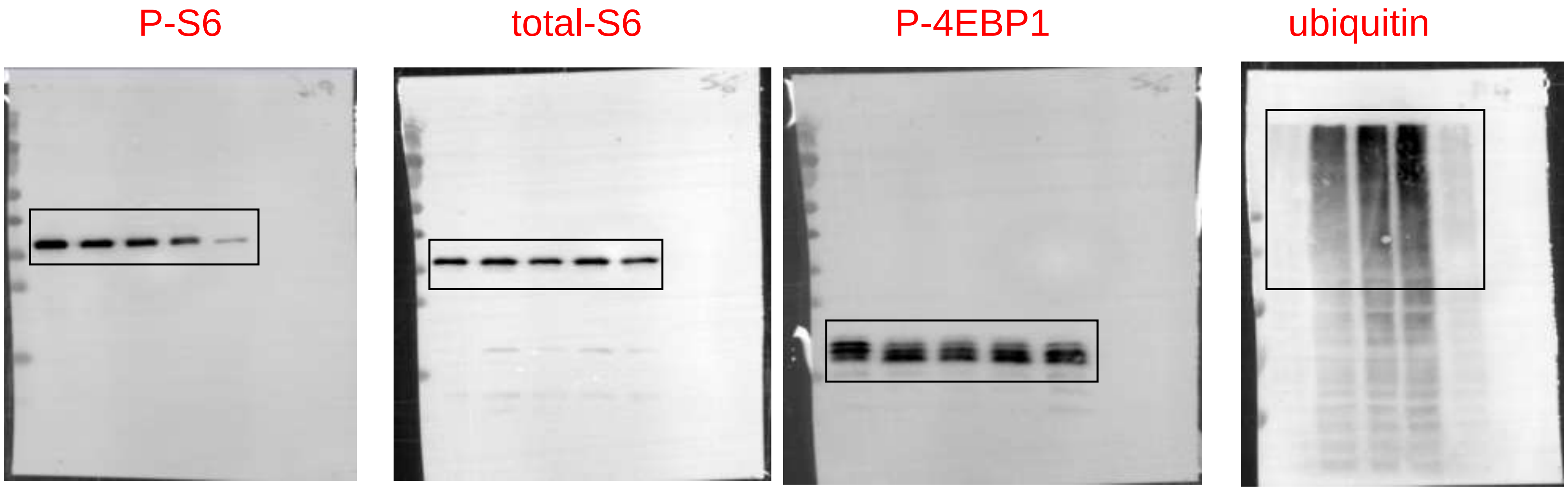

Figure 2B

Cleaved caspase3

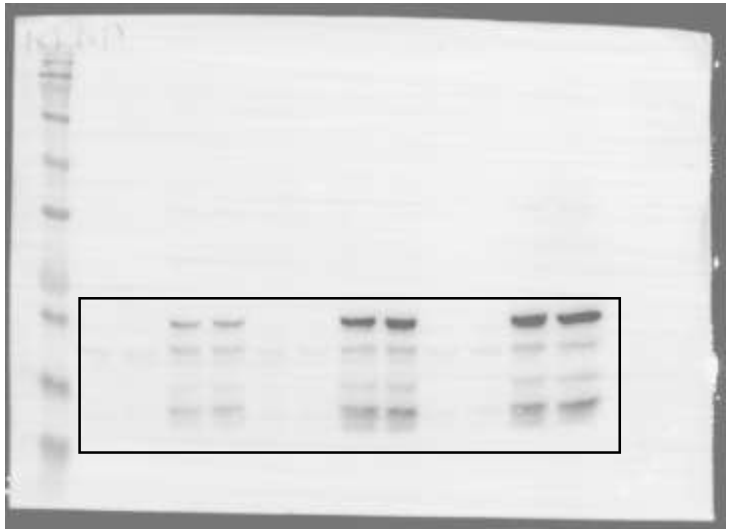

p97

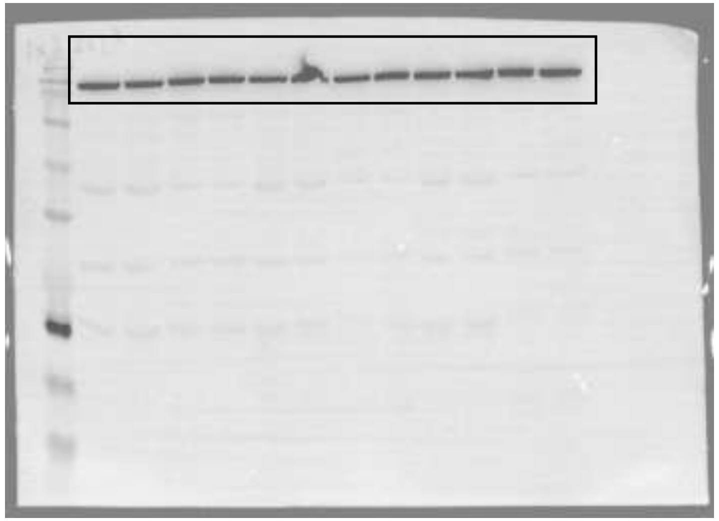

Cleaved caspase3

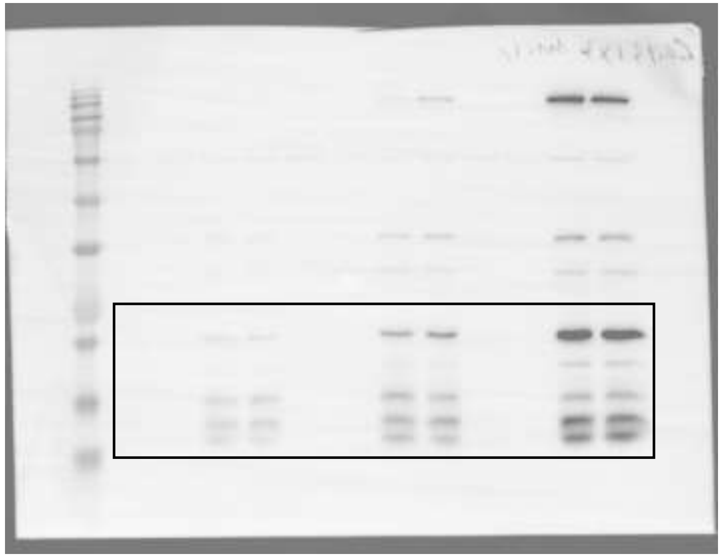

p97

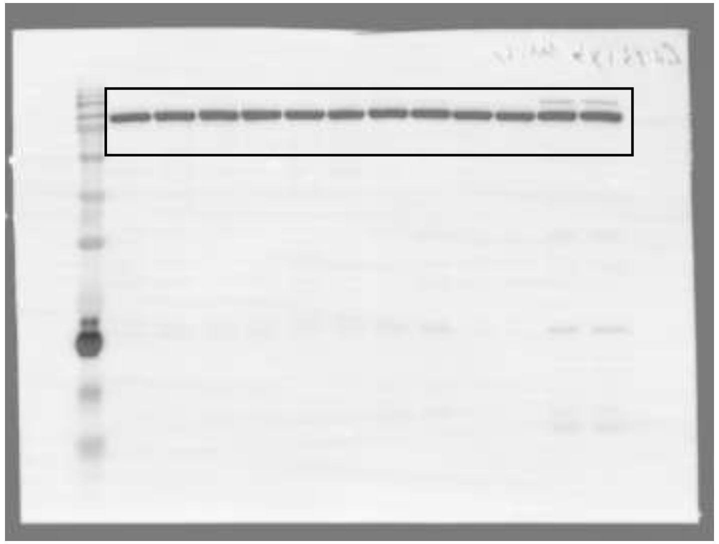

Figure 2D

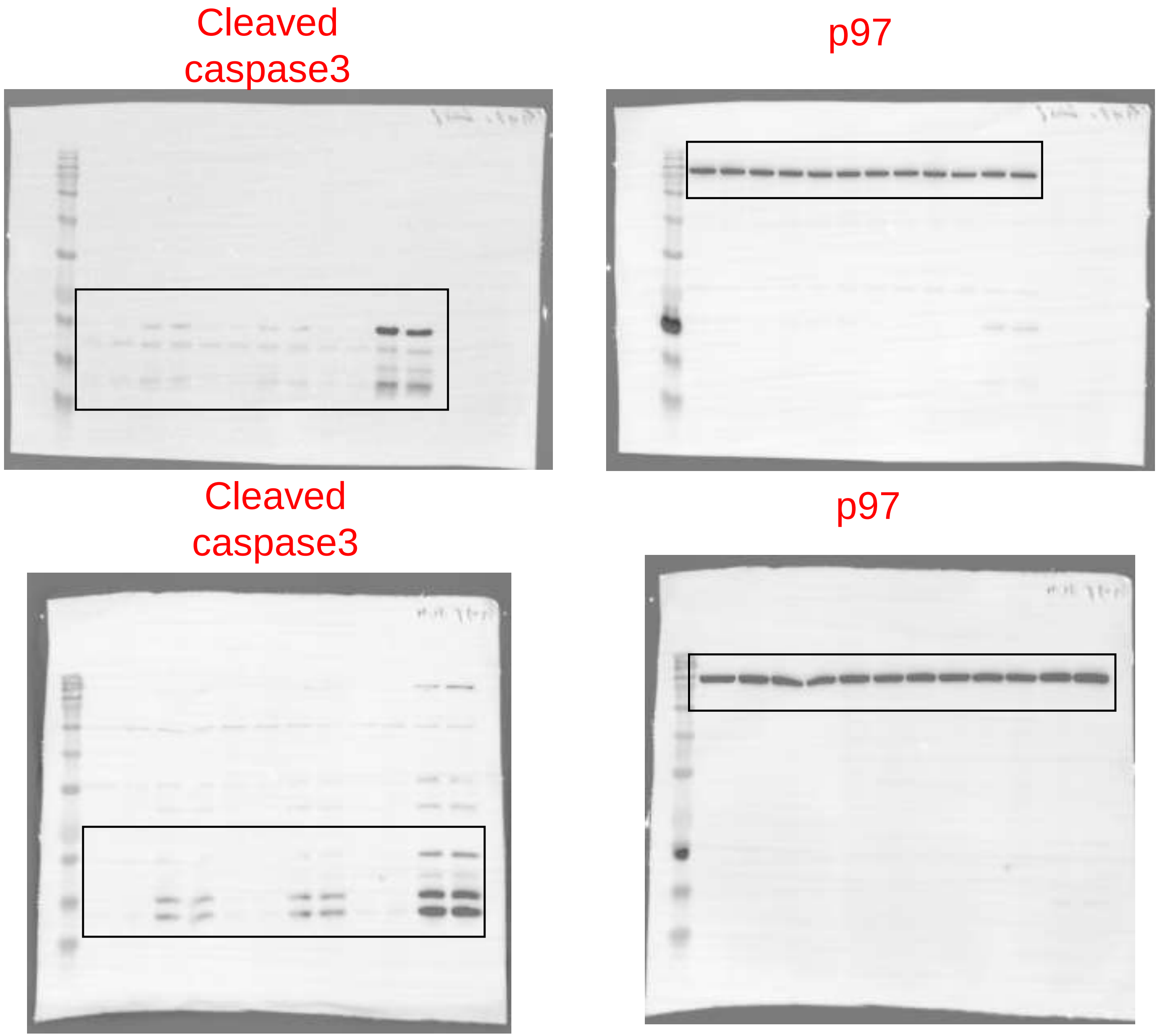

Figure 2F

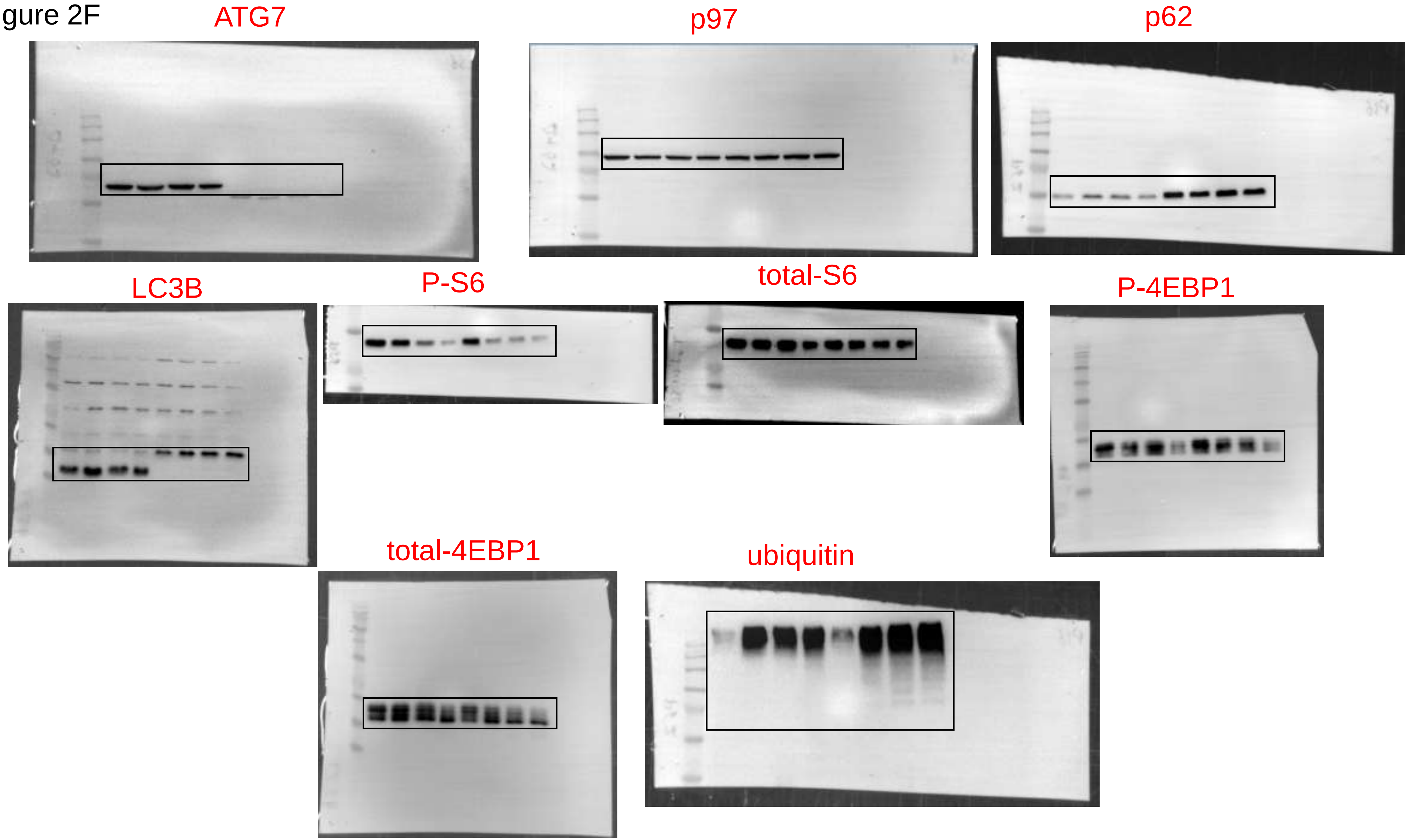

Figure S3A

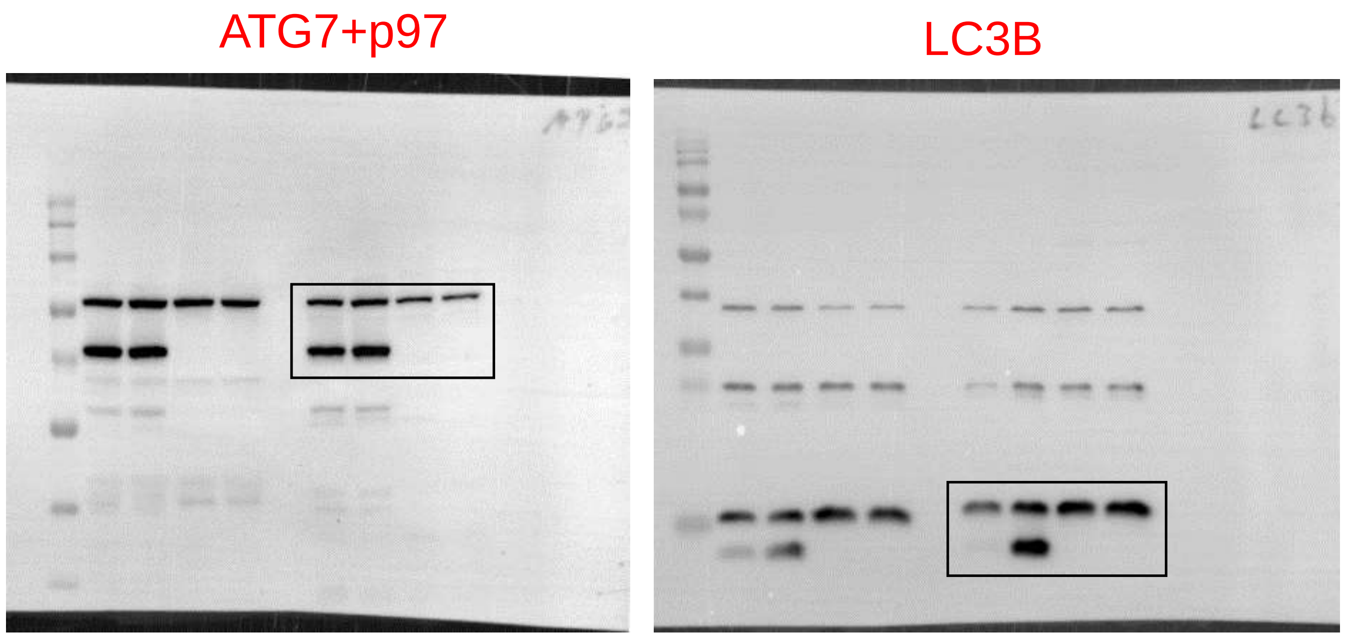

Figure S3B

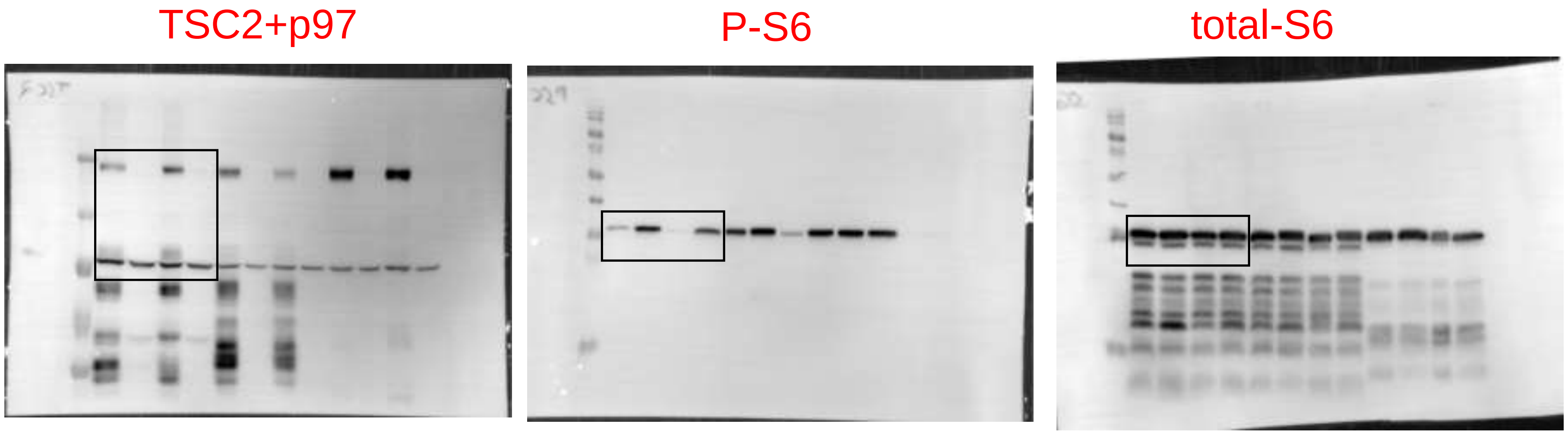

Figure S3C

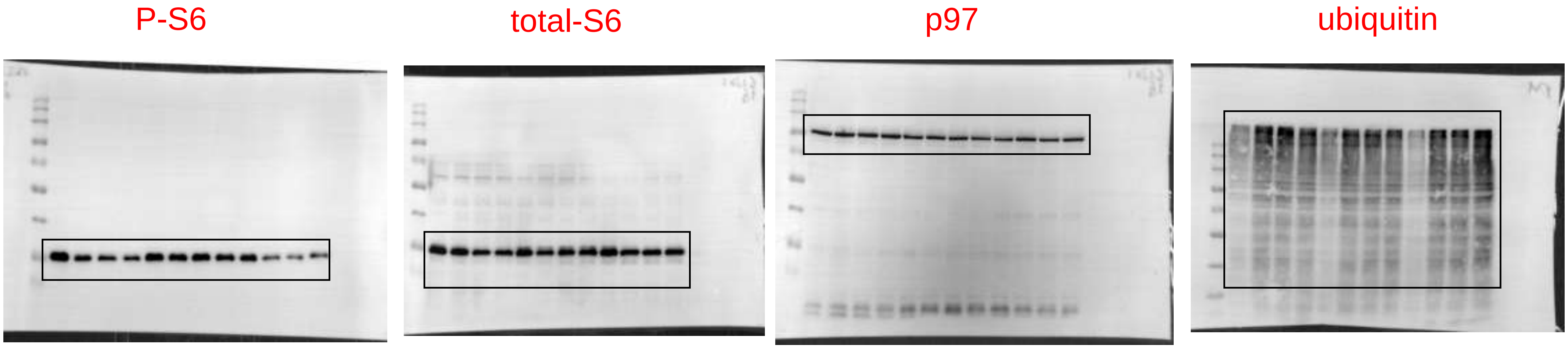

Figure S3E

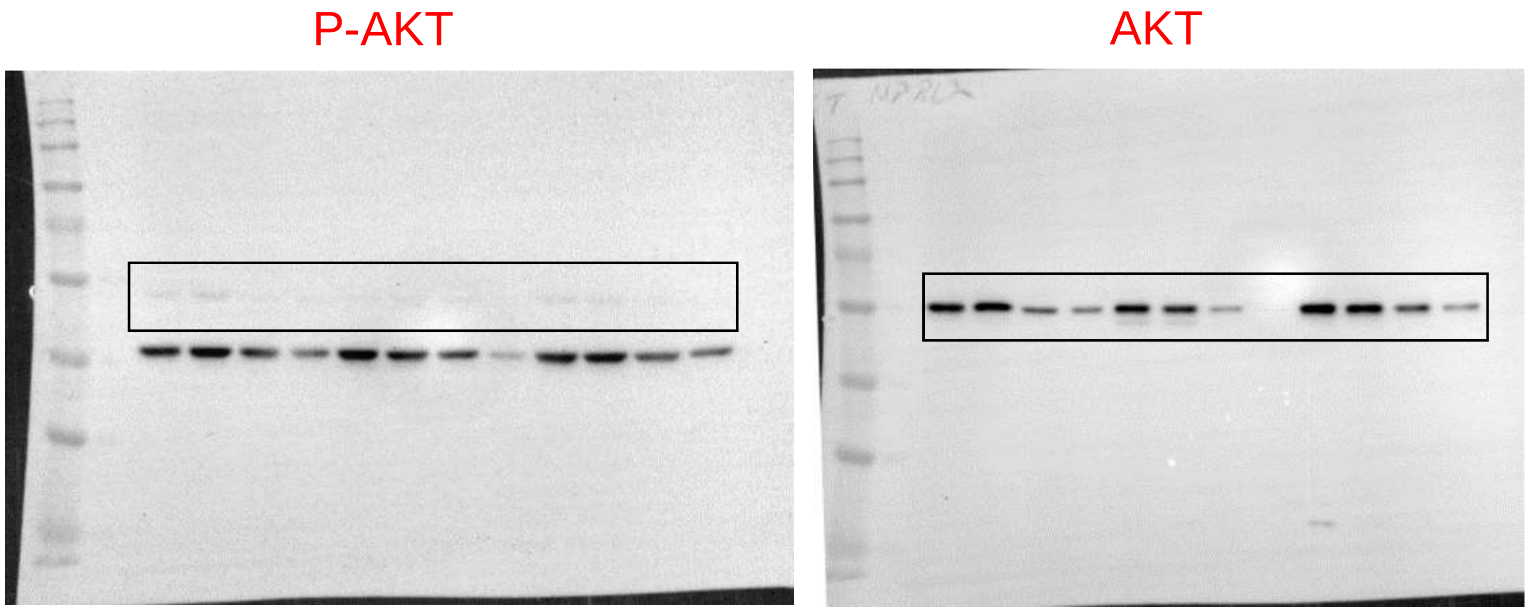

Figure S3F

P-AKT

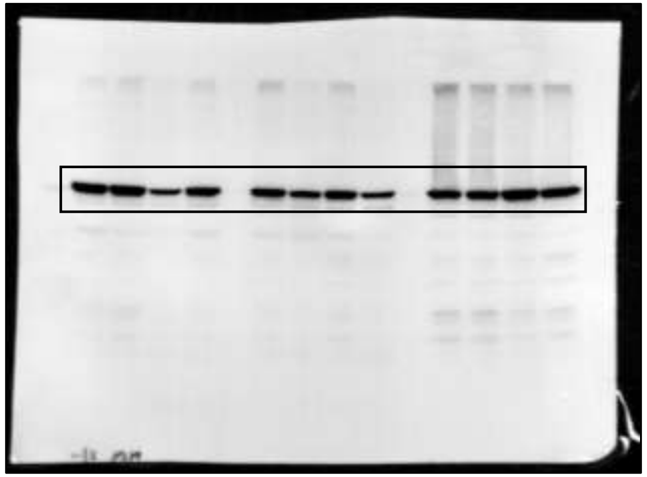

AKT

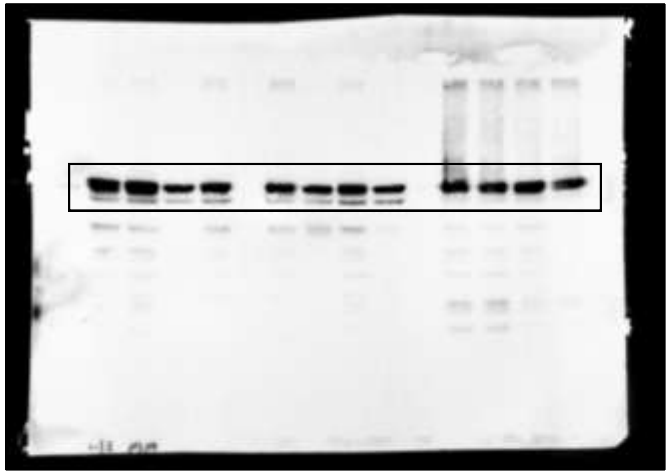

Figure 3A

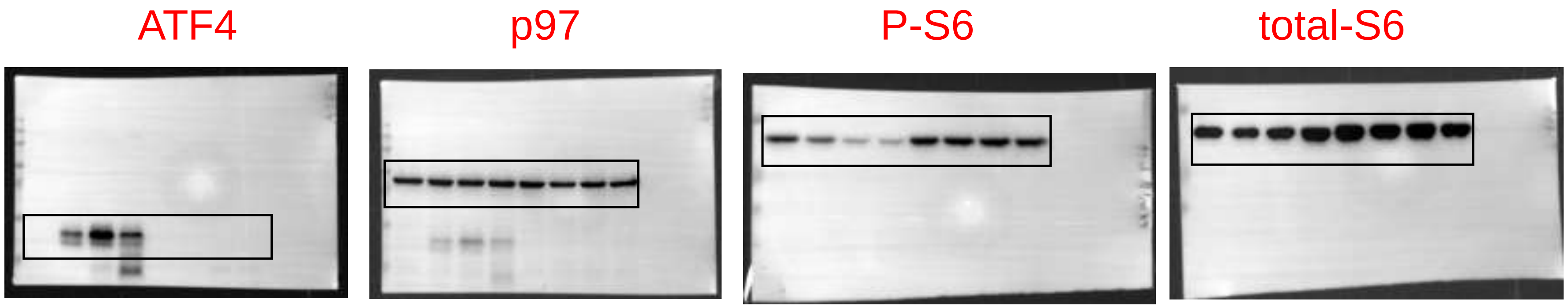

Figure 3B

ATF4+p97

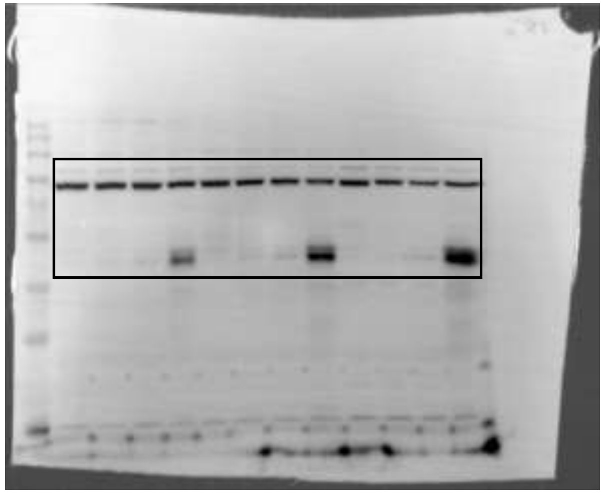

Figure 3C

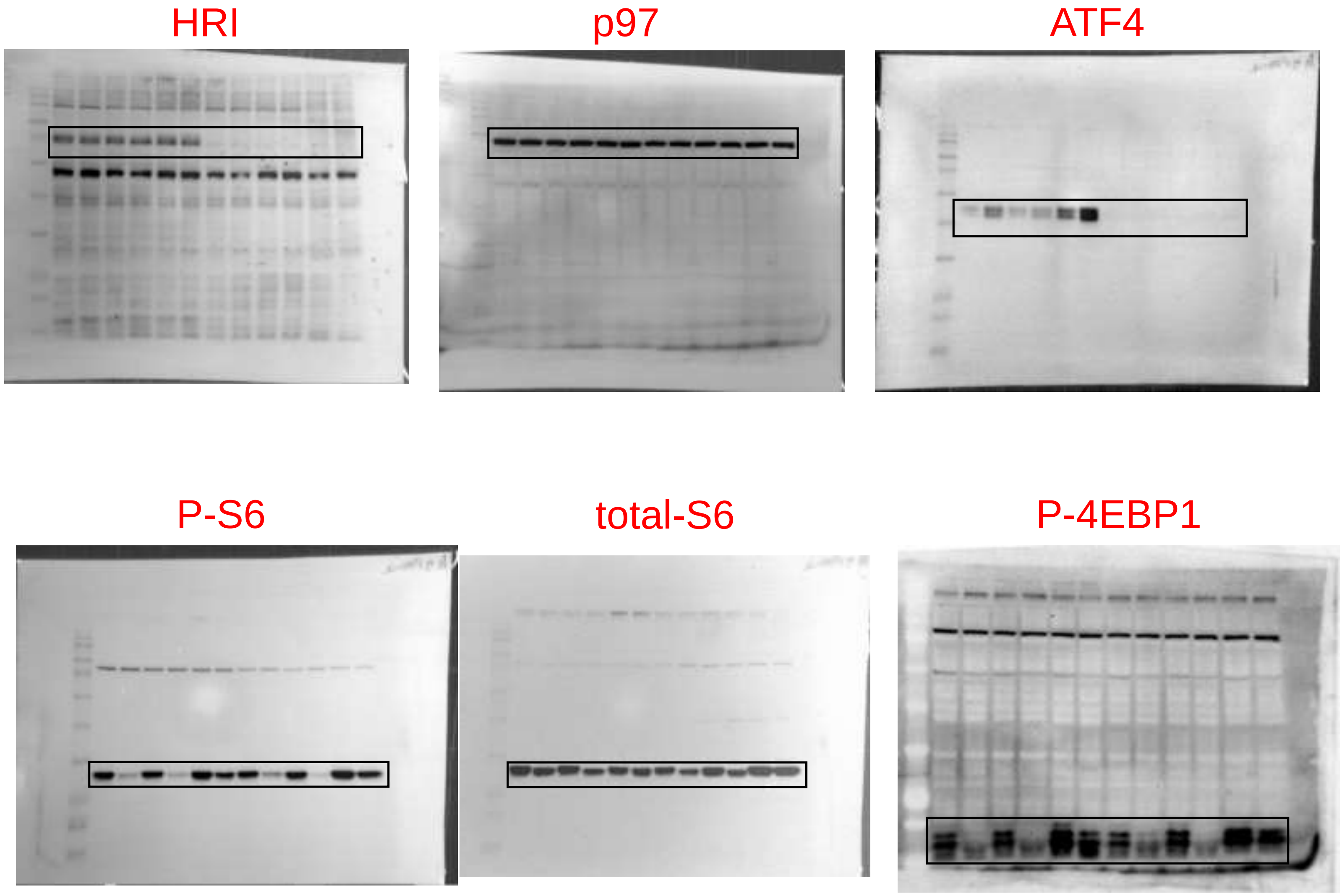

Figure 3D

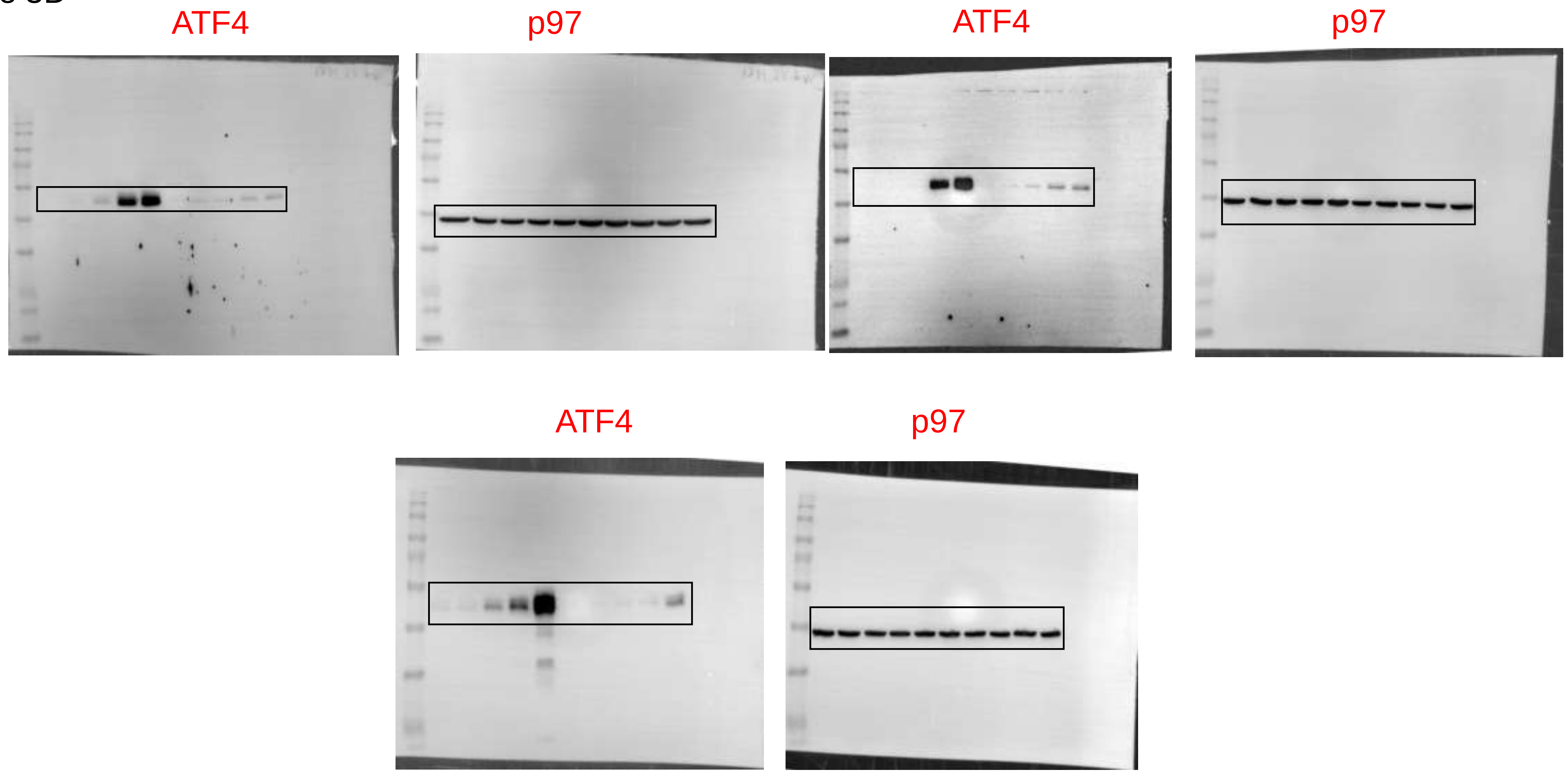

Figure S4A

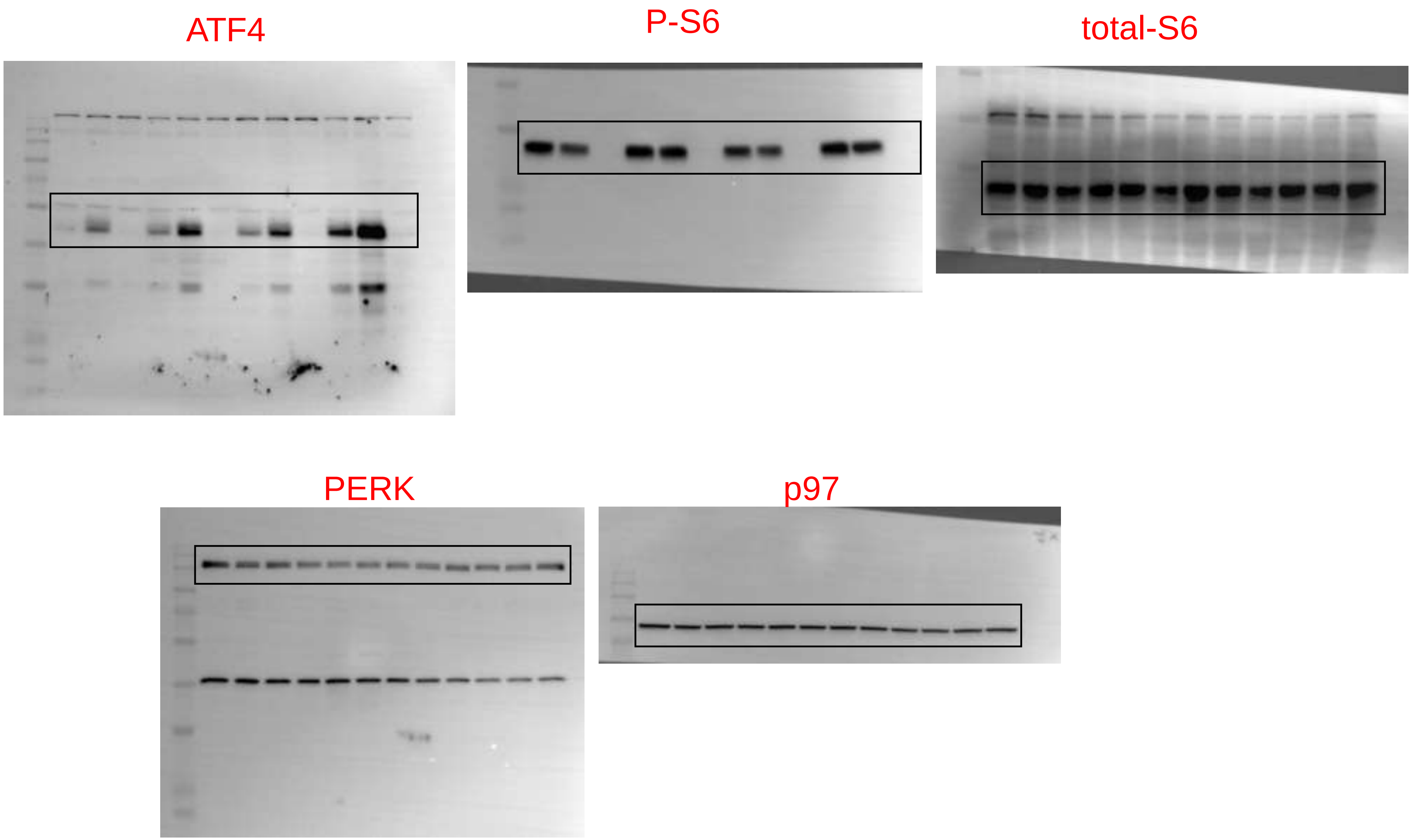

Figure S4B

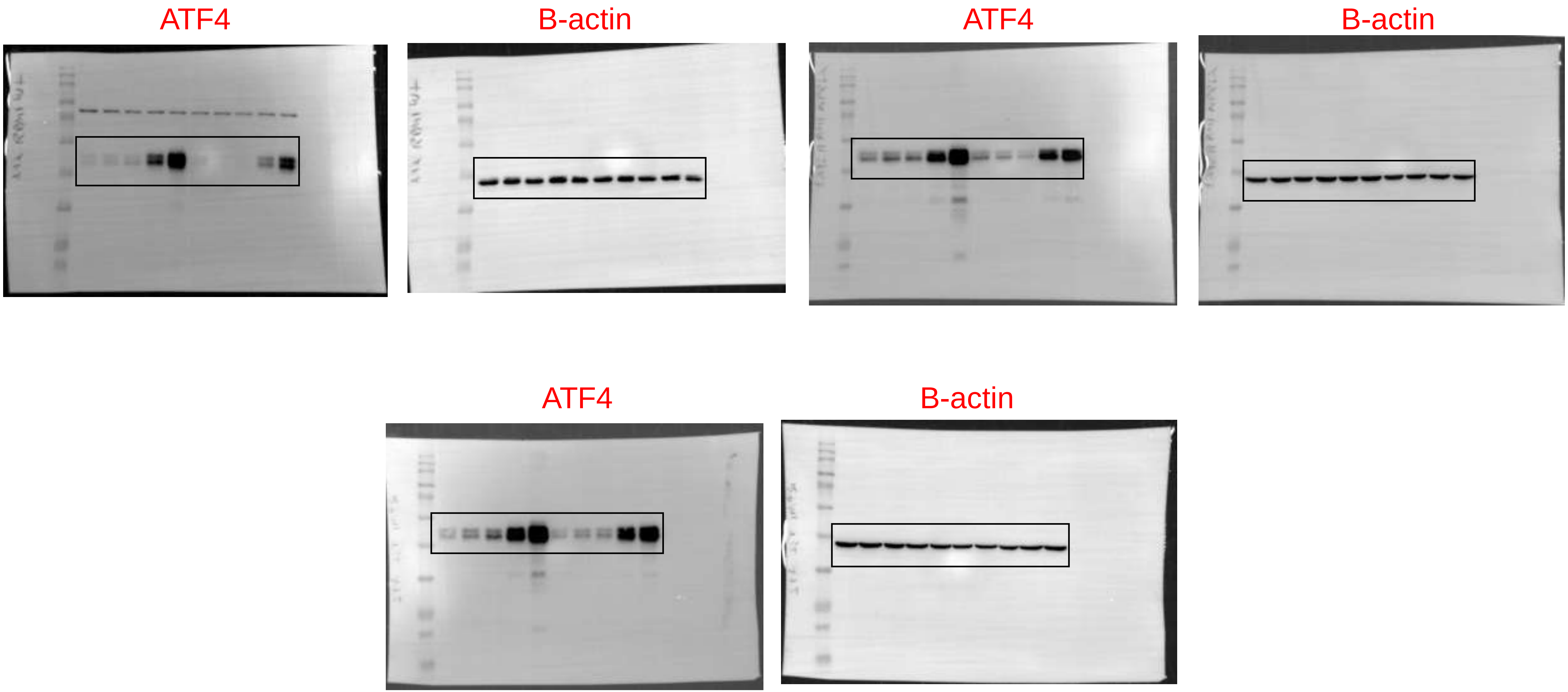

Figure 4A

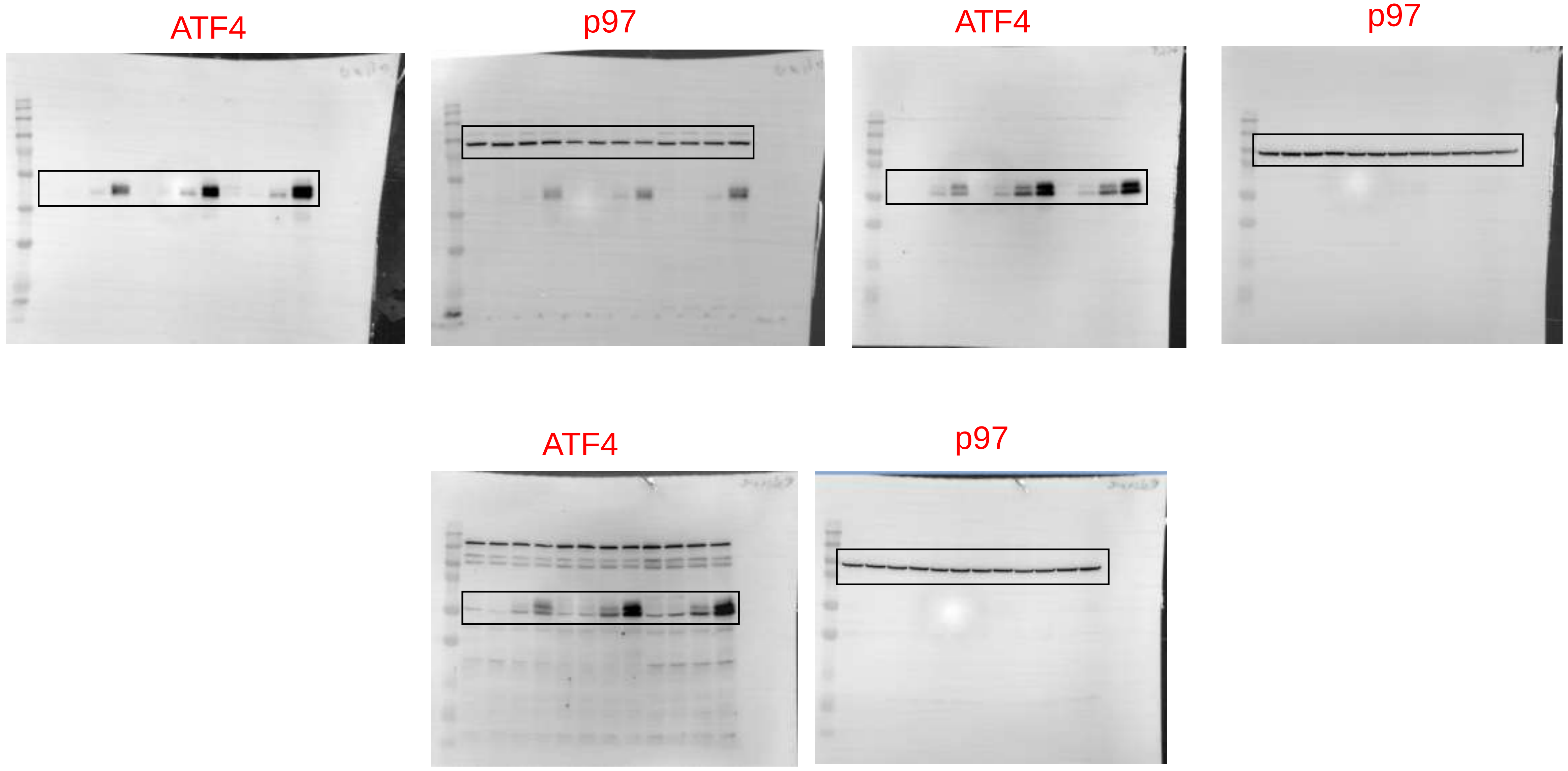

Figure S5A

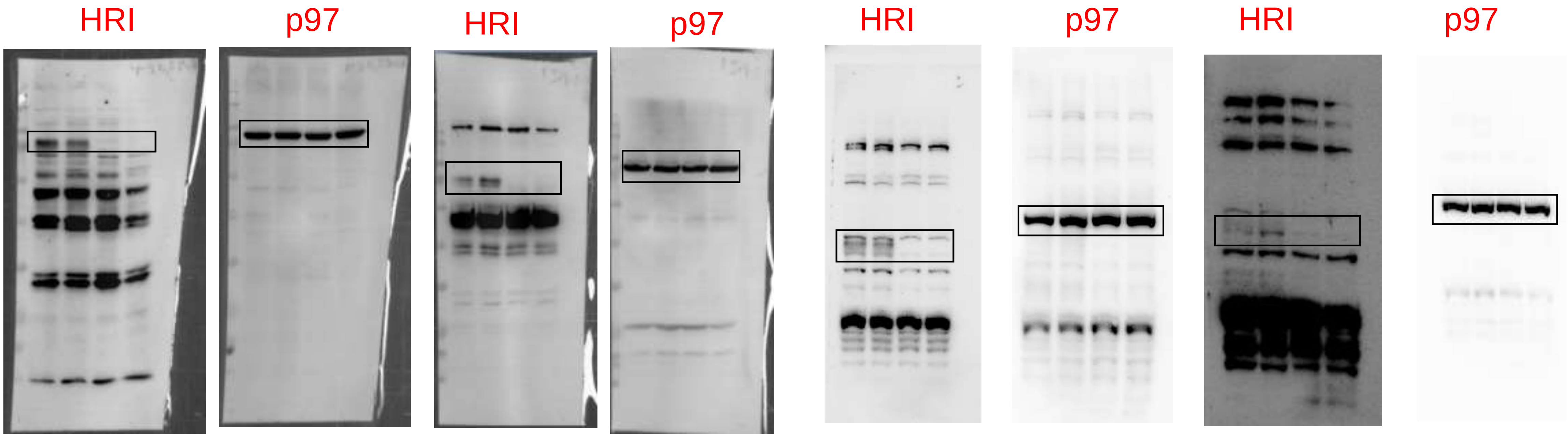

Figure S5C

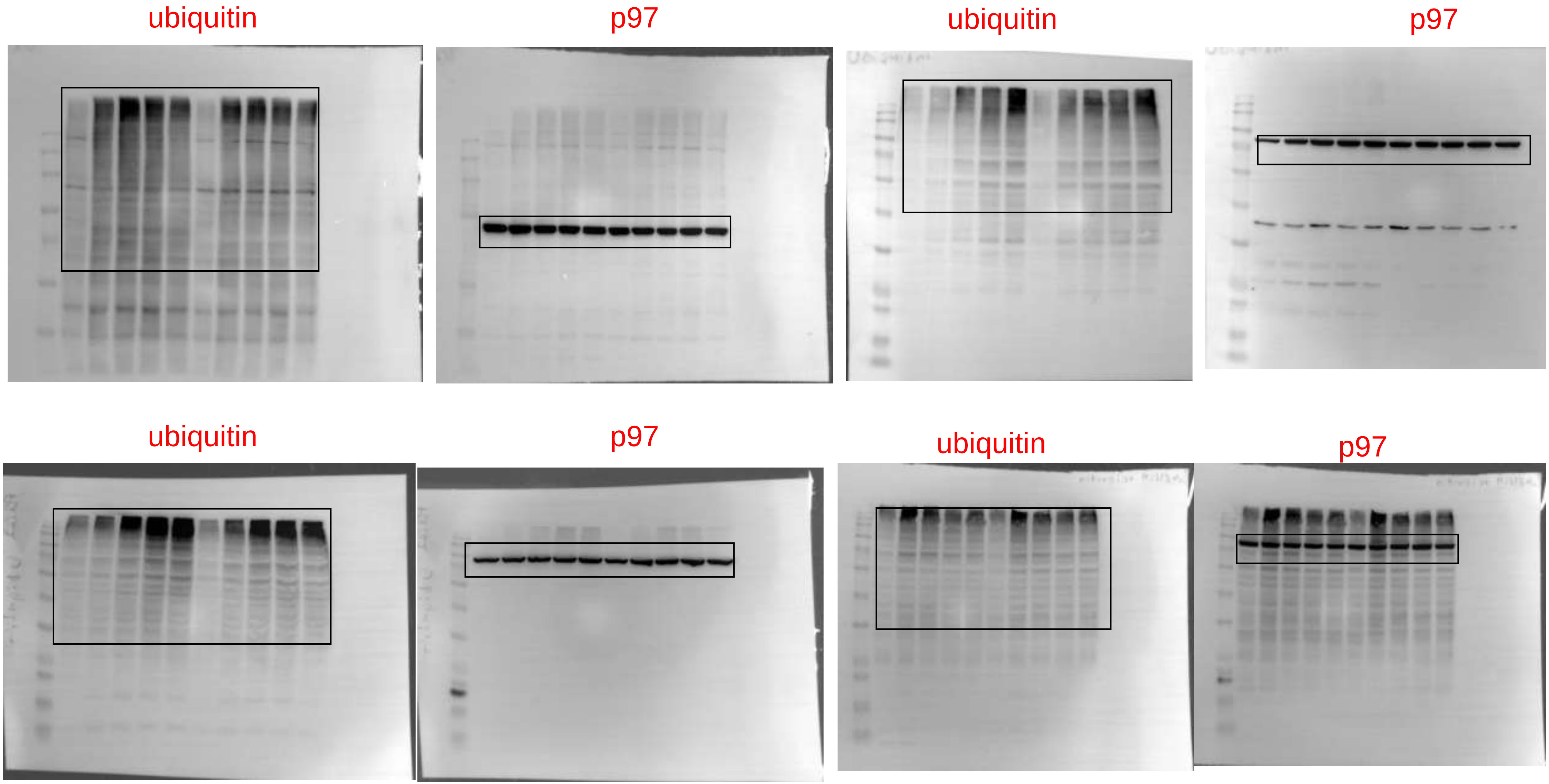

Figure 5B

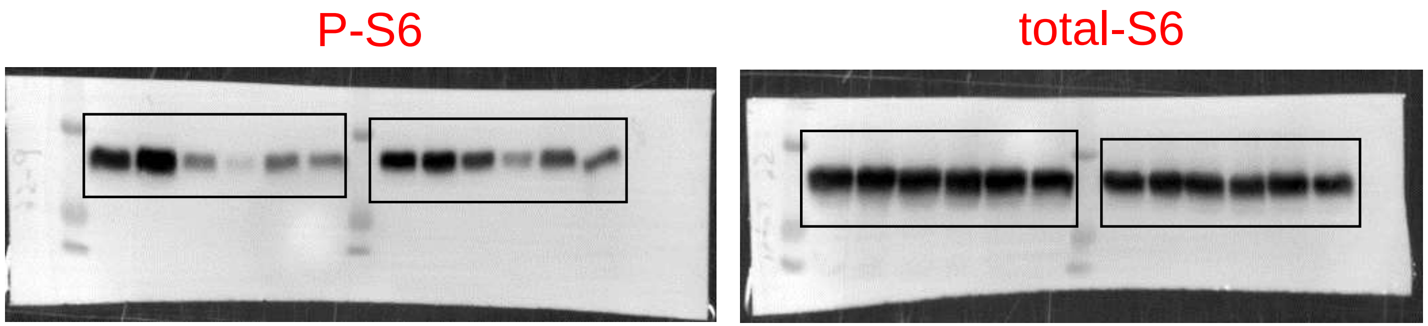

Figure 5C

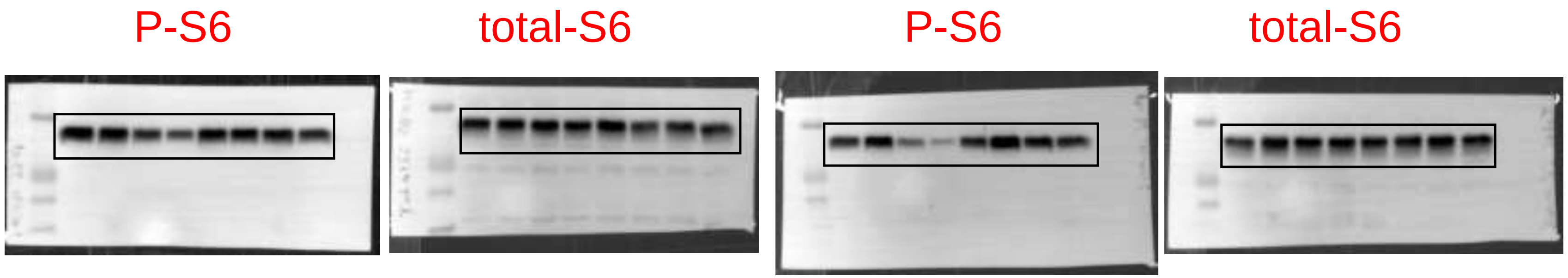

Figure S6A

P-S6

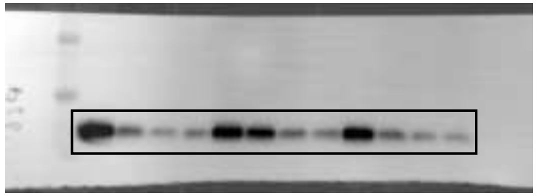

total-S6

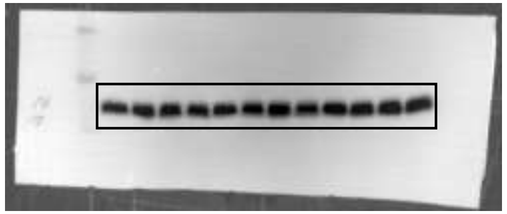

Figure S6B

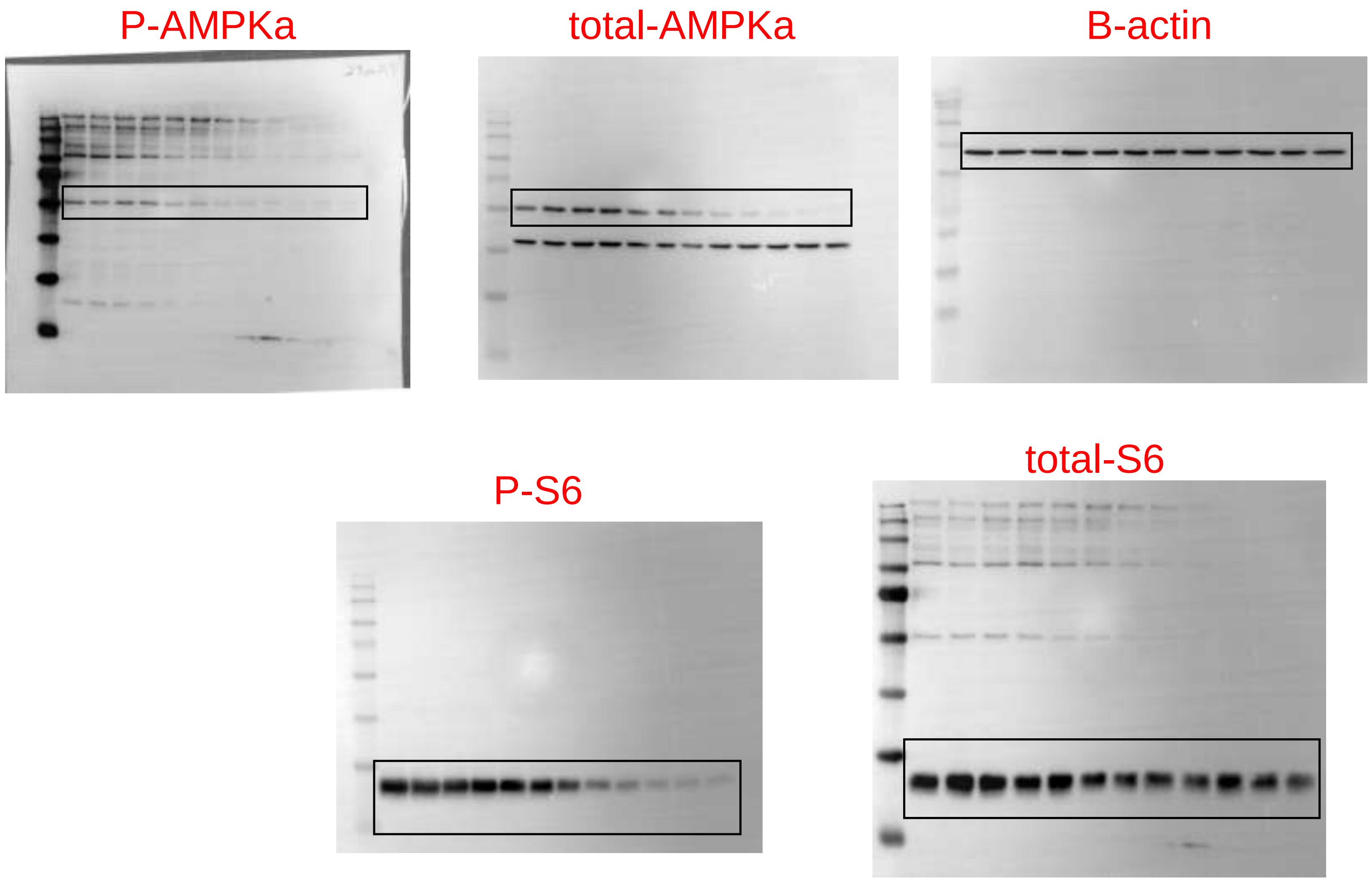

Figure S6C

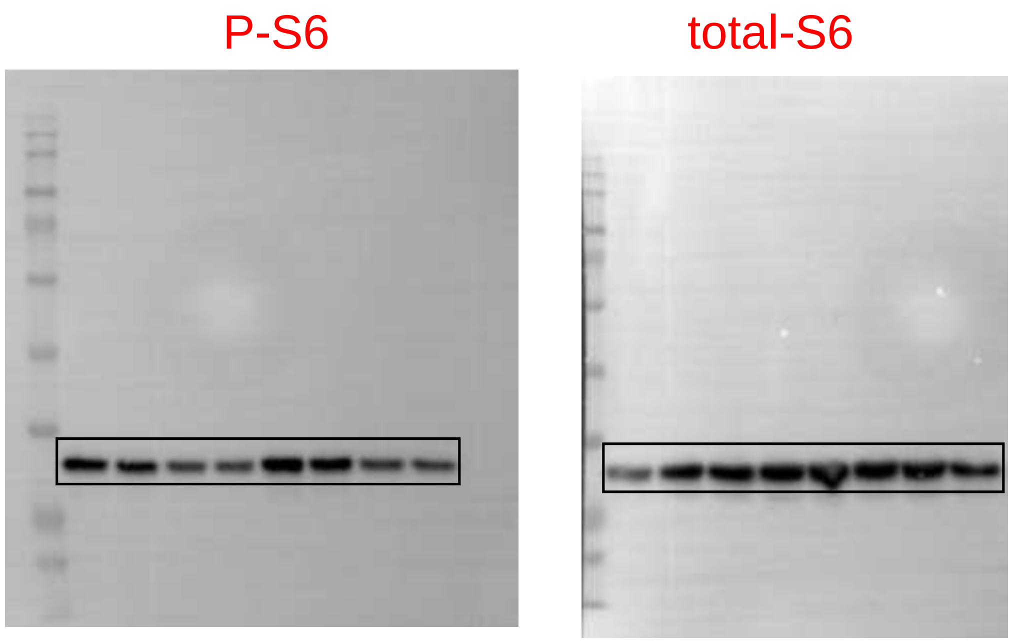

Figure S6D

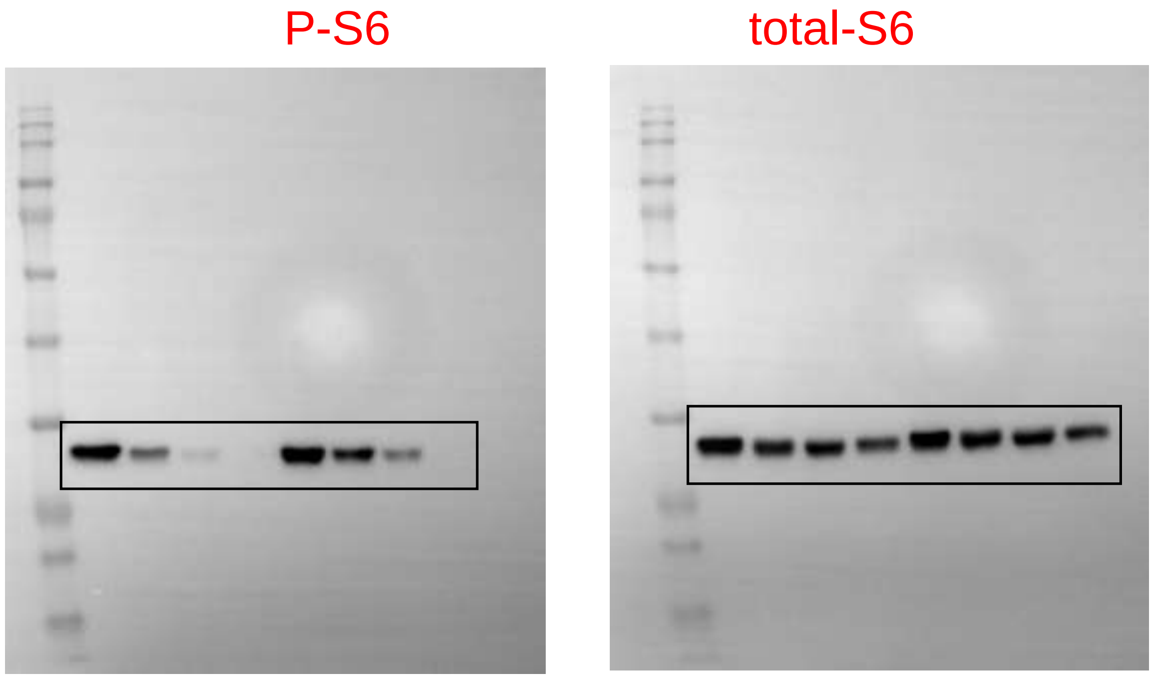

Figure S7A

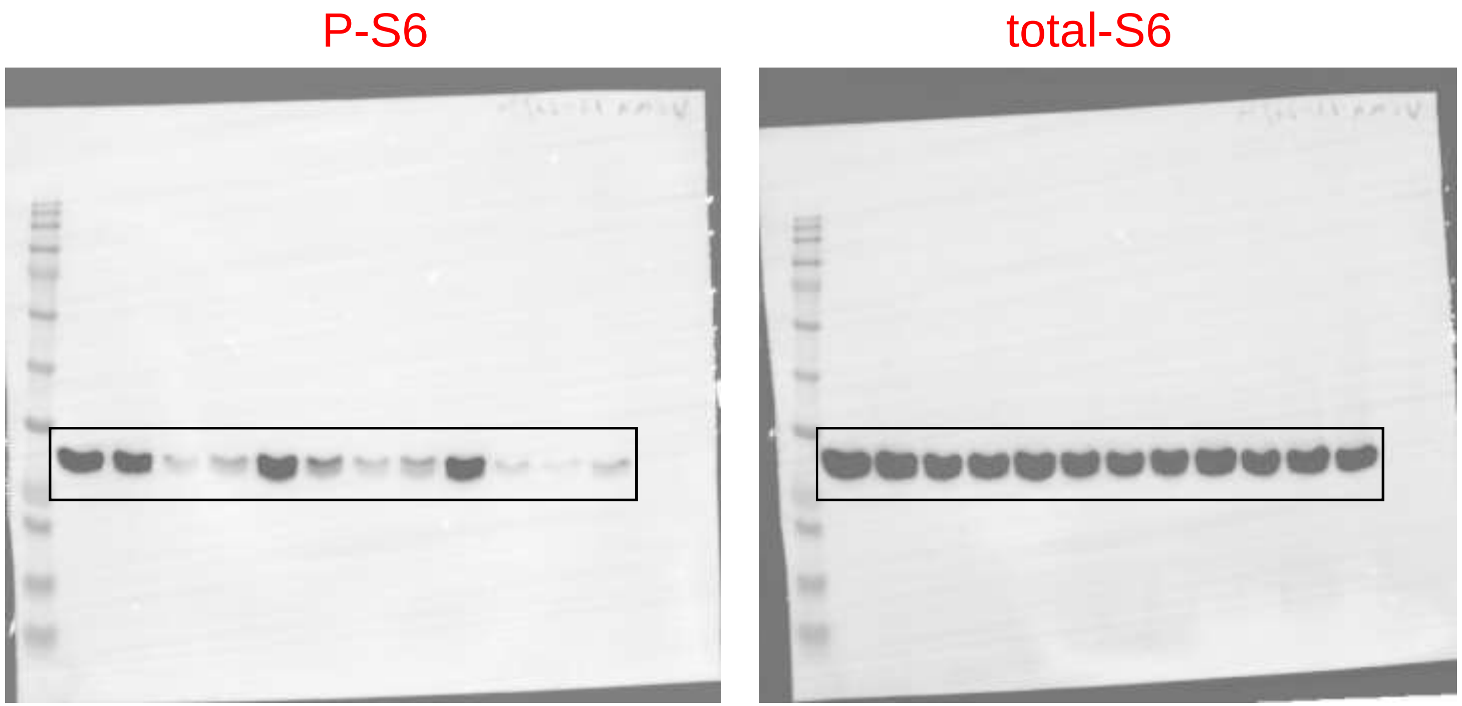

Figure S7B

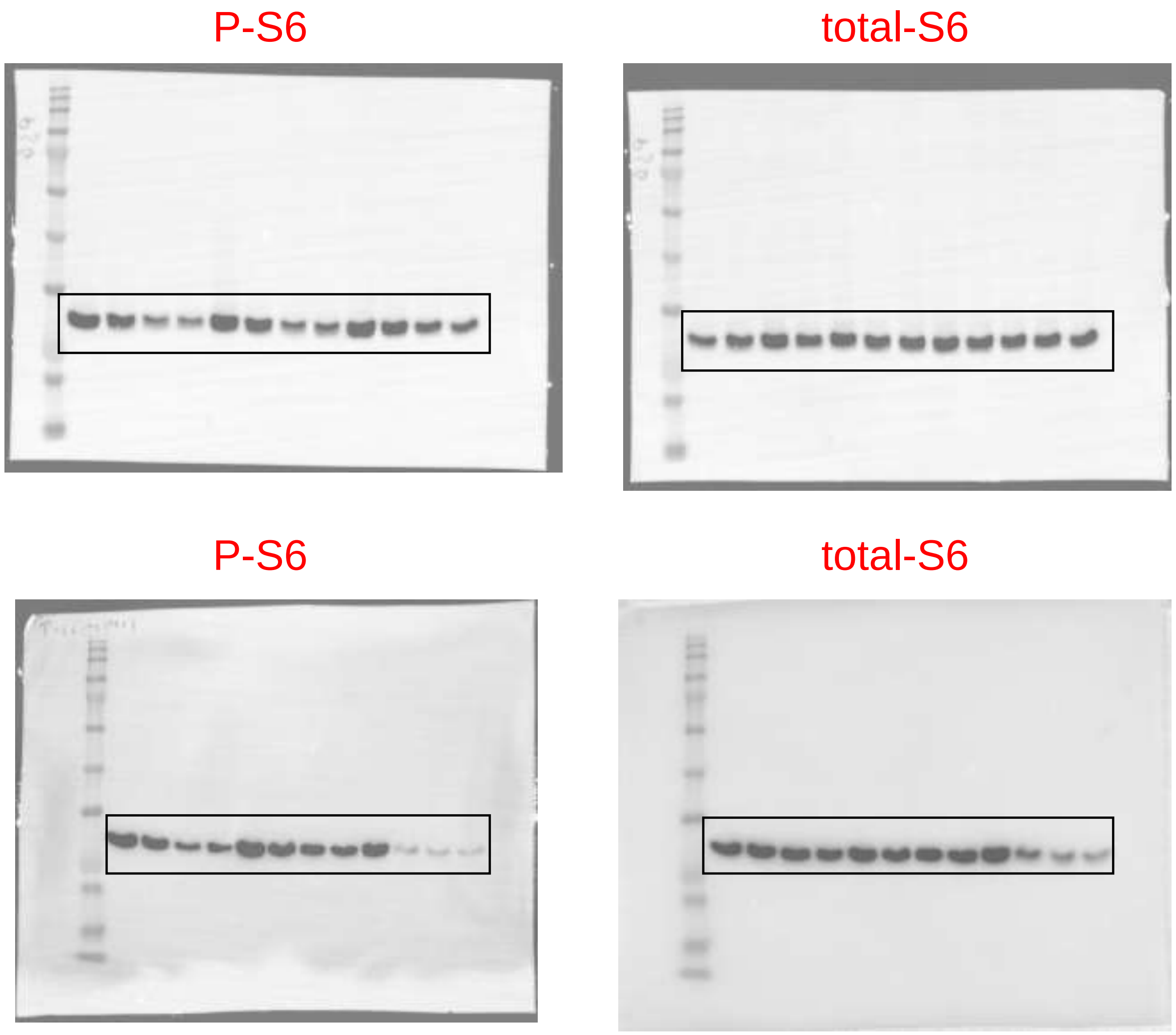

Figure S8A

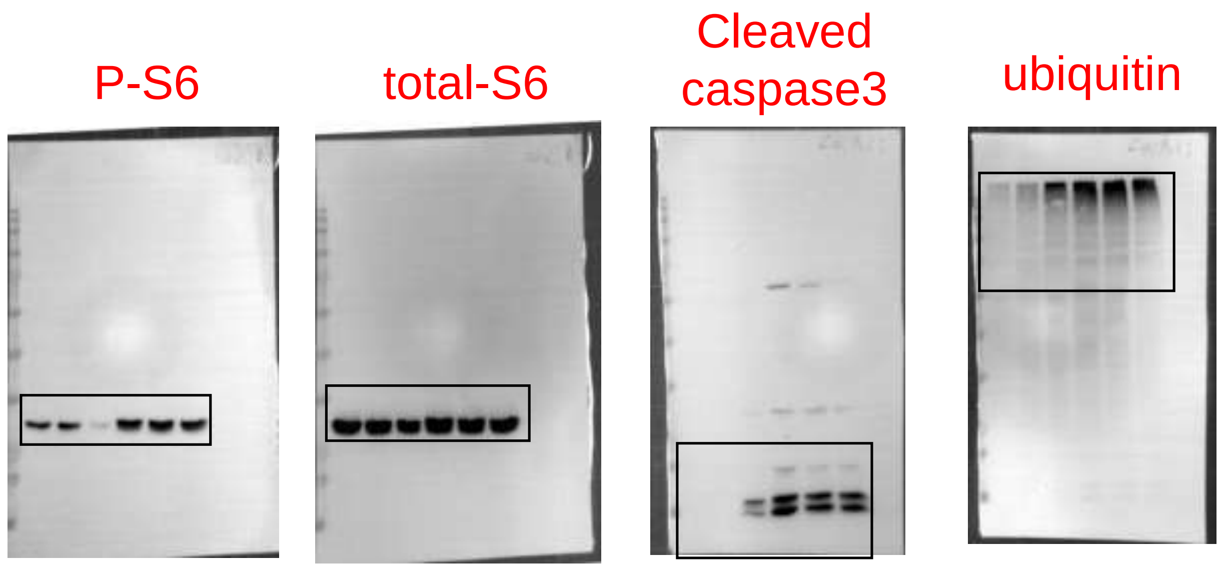

Supplement: Supplementary file 2 — Uncropped immunoblots [file 41419_2022_5421_MOESM2_ESM.pdf]
